# A Novel Strategy to Enhance the Bone Healing Efficacy of Composite Scaffolds via Induction of Cell Recruitment and Vascularization

**DOI:** 10.34133/bmr.0185

**Published:** 2025-04-10

**Authors:** Jeong In Kim, Thi Thu Trang Kieu, Jeong-Chae Lee

**Affiliations:** ^1^ Department of Orthopaedic Surgery, CHA Bundang Medical Center, CHA University School of Medicine, Seongnam 13488, South Korea.; ^2^ Department of Bioactive Material Sciences, Research Center of Bioactive Materials, Jeonbuk National University, Jeonju 54896, South Korea.; ^3^ Cluster for Craniofacial Development and Regeneration Research, Institute of Oral Biosciences and School of Dentistry, Jeonbuk National University, Jeonju 54896, South Korea.

## Abstract

This study devised a novel strategy to develop a functionally improved scaffold that enhances the healing of large bone defects via synergistic activation of vascularization and cell recruitment. To this end, we fabricated round and ring-shaped silk fibroin/*Broussonetia kazinoki* (SFBK) composite scaffolds. The round scaffolds had a diameter of 1.5 mm, and the ring-shaped scaffolds had a 6-mm diameter with a 1.5-mm hole in the center. All scaffolds had a 3-mm thickness. A portion of round SFBKs was cross-linked with stromal cell-derived factor 1 (SDF-1), and ring-shaped scaffolds underwent in vitro angiogenic stimulation, in vivo vascularization, or both. These scaffolds were assembled by fitting a round SFBK into the center of a vascularized SFBK scaffold before implantation into a rat model with critical-sized calvarial defects. Implantation with puzzle-fitted scaffolds promoted bone regeneration, and the scaffold that underwent both SDF-1 immobilization and vascularization processes showed the greatest efficacy in the healing of defects. The bone healing efficacy of puzzle-fitted scaffolds involved their ability to stimulate microvascular network formation, collagen synthesis, and stem cell recruitment at defects. *B. kazinoki*-released calcium ions also participated in synergistic bone regeneration. These results suggest that the strategy of fitting SDF-1-linked SFBK into a vascularized ring-SFBK scaffold is useful in recruiting multipotent stem cells via newly formed blood vessels toward the center of scaffolds. This induces balanced and uniform bone regeneration. Overall, this study highlights the needs of calcium release, neovascularization, and stem cell recruitment for synergistic enhancement of bone regeneration.

## Introduction

The prevalent challenge facing tissue engineering for the treatment of large bone defects is the maintenance of the controlled angiogenic and vasculogenic activation required for bone regeneration [1,2]. The principle of successful bone tissue engineering relies on the scaffold’s ability to guide rapid and sufficient vascularization in target tissues [3]. Over the past decades, researchers have utilized tissue engineering techniques with a variety of strategies to enhance vascular development. There are 4 classes of common vascularization strategies: direct stimulation by proangiogenic factors, in vitro prevascularization, prevascularization by in vivo implantation, and miscellaneous strategies [4,5].

Implantation of preformed vascular networks may enhance the ingrowth and perfusion of host vessels and allow successful regeneration of defective tissues [6,7]. However, in vitro vascularization requires the right balance of cell types within the system and is difficult to manage with multiple cell types [8]. In vitro vascularization also has limited success when large scaffolds are implanted to induce uniform bone tissue placement over a large defect. This limitation is closely associated with decreased angiogenic potential in the center of the scaffolds due to the lack of newly formed blood vessels during tissue regeneration. However, in vivo prevascularization is uncomplicated, and the use of an in vivo prevascularized scaffold may address some practical limitations such as suboptimal integration of the biostructure within the host tissue [8–10]. This strategy has been successfully utilized for the repair of neural tissue and large-sized bone defects, in which guided vascularization in and around the scaffolds is the key event [11]. Consequently, the use of an in vivo vasculogenic strategy might overcome a limitation derived from in vitro vascularization and enhance scaffold-induced regeneration at target tissues.

Stem-cell-based strategies are another promising approach to treating large bone defects [12,13]. However, low viability, genetic instability, and aberrant differentiation of transplanted stem cells and long-term efforts for cell expansion are major challenges in advancing stem cell therapies [14]. An alternative strategy for tissue engineering for large bone defects is the design of scaffolds that stimulate new bone formation and remodeling mechanisms through the recruitment of endogenous stem cells into the center of scaffolds implanted at injured sites. For this, the use of stromal cell derived factor-1 (SDF-1), a chemoattractant, is a conventional approach in guiding tissue repair without an external source of cells [15,16]. Overall, this approach depends on the development of target-specific scaffolds that mobilize host stem cells. This is affected by temporally and spatially controlled release of biological signals inside scaffolds in target tissues.

Efficient construction of large bone tissues requires the fabrication of bioactive scaffolds that promote in vivo formation and maturation of vascular networks and induce the recruitment and differentiation of stem cells. We devised a novel modular assembly strategy to develop a bioactive scaffold that enhances the healing of large bone defects via the synergistic activation of vascularization and stem cell recruitment. For this work, we chose silk fibroin (SF) and *Broussonetia kazinoki* (BK), because SF-mixed BK (SFBK) scaffolds triggered proliferation and osteogenic differentiation of multipotent stem cells and in vivo bone regeneration as efficient and biocompatible cell carriers [17]. BK is also used as a traditional medicine to treat burns and acne and contains various biological components [18]. In this study, we fabricated SFBK scaffolds (6-mm diameter/3-mm thickness) in 1 of 2 forms, round or ring shaped. The round scaffolds were 1.5 mm in diameter, and ring-shaped (ring-SFBK) scaffolds were 6 mm in diameter with 1.5-mm holes in the centers. A portion of the round SFBK scaffolds underwent 1-ethyl-3-(3-dimethylaminopropyl)carbodiimide (EDC)/*N*-hydroxysuccinimide (NHS) activation to construct SDF-1-cross-linked (SFBK-S1) scaffolds. A portion of the ring-SFBK scaffolds underwent an in vivo vascularization process to produce vascularized ring-SFBK (VR-SFBK) scaffolds. Scaffolds were combined using the puzzle-fitting process by placing a round SFBK into the center of a ring-SFBK scaffold. Implantation with the puzzle-fitted scaffolds promoted bone regeneration in a rat model of critical-sized calvarial defects. Specifically, the implantation of an SFBK-S1-fitted VR-SFBK scaffold showed the greatest efficacy on bone repair, involving increases in microvascular networks, collagen synthesis, and stem cell recruitment. Additionally, the results of this study suggest that calcium ions are released from SFBK scaffolds and participate in synergistic bone regeneration. Overall, this study highlights the need for neovascularization as a tool for stem cell mobilization for tissue regeneration and the value of vascular tissue engineering in stem cell applications.

## Materials and Methods

### Preparation of SF solution and BK powder

For removal of sericin, pieces of silkworm cocoons were boiled in a solution containing 0.02 M Na_2_CO_3_ (Showa Chemical Industry Co., Japan) for 30 min. Degummed fibers were removed by washing the cocoon pieces with distilled water. After being dried in a fume hood overnight, the dried silk cocoons were dissolved in a 9.3 M lithium bromide monohydrate solution (LiBr; Kanto Chemical Co., Japan) at 60 °C for 4 h to yield an SF solution. Residual LiBr in the SF solution was removed using a dialysis tube (SnakeSkin, Thermo Fisher Scientific, Waltham, MA, USA) with a molecular weight cutoff of 3.5 kDa for 72 h in distilled water. Finally, the concentration (8%, w/v) of SF in the solution was confirmed by gravimetric analysis. The solution was stored at 4 °C until further use. BK (10 g) was boiled in 0.1 M NaOH for 4 h, and then the nonfibrous material of the peeled BK was removed. Boiled BK was washed in distilled water to remove residual NaOH. After complete freeze-drying, BK was freeze-pulverized and manufactured in the form of powder using a freeze-milling machine (SPEX SamplePrep Freezer/Mill, Cole-Parmer, NJ, USA).

### Fabrication of SF and SFBK scaffolds

A pure SF solution and a solution in which BK powder was dispersed in the SF solution were poured into a 96-well plate and lyophilized overnight. The lyophilized scaffolds were cross-linked in methanol and rinsed with distilled water for 1 h. The cross-linked scaffolds were evenly cut into 3-mm-thick/6-mm-diameter sections to yield SF and SFBK scaffolds, respectively. Additionally, round and ring-shaped SFBK scaffolds were created for cross-linking with SDF-1 and in vivo vascularization, respectively, by introduction of central 1.5- or 4-mm holes.

### Cross-linking or loading of SFBK scaffolds with proteins

Aspartic and glutamic acids in SF, which carry carboxylic acid side chains available for carbodiimide chemistry, are used to immobilize SDF-1 through a direct reaction with the primary amines of the protein after the EDC/NHS activation process. In brief, round SFBK scaffolds (1.5-mm diameter) were soaked in 2-(*N*-morpholino)ethanesulfonic acid (MES) buffer (5 mM, pH 5.5 to 6.0) for 30 min, and each of the scaffolds was immersed in fresh MES buffer solution (5 μl) containing 2 μg of SDF-1. The scaffolds were incubated in 50 mM EDC and 25 mM NHS in MES buffer for 20 h to yield the SDF-1-cross-linked scaffolds, SFBK-S1. After a brief washing with distilled water, SFBK-S1 scaffolds were dried and stored at room temperature before use. Alternatively, each of the ring-shaped SFBK scaffolds (6-mm diameter with a 1.5-mm hole) was loaded with phosphate-buffered saline (PBS; 20 μl) including 2 μg of angiopoietin-1 (Ang1) immediately before the use for in vivo vascularization.

### XRD and XPS analyses

X-ray diffraction (XRD; X’Pert Pro, Malvern Panalytical, UK) was performed to analyze the composition of BK powder and the form of calcium ions in SFBK scaffolds. XRD patterns were measured with a Bruker (D8 Advance) x-ray diffractometer with a Cu Kα radiation source over a range of 2*θ* angles (10° to 60°) at a scan speed of 3°/min. The interaction of several forms of calcium ions with SFBK scaffolds was analyzed by x-ray photoelectron spectroscopy (XPS; Kratos Axis Ultra DLD spectrometer). High-resolution XPS spectra of Ca 2p, O 1s, and C 1s were collected using monochromatic Al Kα (1,486.58-eV) radiation at an analyzer pass energy of 50 eV.

### Morphostructural characterization

The morphological characteristics of the surface architecture and porosity of scaffolds were observed by scanning electron microscopy (SEM; JSM-5900LV, JEOL, Japan). The distribution and characterization of the mineral phase in the BK, SF, and SFBK scaffolds were investigated through SEM–energy-dispersive spectroscopy (EDS) analysis. Elemental mapping of the element Ca showed perfect overlap in SF scaffolds, confirming that Ca was evenly distributed within the scaffolds.

### Mechanical and degradation tests

The compression and degradation properties of ring-shaped SFBK scaffolds were evaluated before and after the puzzle-fitting process. In this assay, compression testing was performed in dry and wet conditions using Oriental Testing Machine (Oriental Testing M/C, version 12.7.2) by manually adjusting the load cell to a position about 1 mm on the specimen. A preload step was initiated using software to bring the crosshead in direct contact with the specimen. A 500 kgf/cm^2^ load cell was employed to detect changes and the displacement of the crosshead from its original position. A crosshead speed of 2 mm/min was adjusted for the mechanical test. To test the degradation property, ring-shaped SFBK scaffolds subjected to the puzzle-fitting process or not were weighed (*W*_0_) and placed in plastic flasks with 15 ml of PBS at 37 °C following incubation for various times (0 to 30 d). At several time points after incubation, scaffolds were washed with distilled water 3 times and dried. The degraded weight (*W_t_*) of PBS-incubated scaffolds was calculated and expressed as % of the initial scaffold weight (*W*_0_).

### Determination of calcium ions and SDF-1 contained in or released from SFBK scaffolds

The concentration of calcium ions contained in or released from SFBK scaffolds was determined by inductively coupled plasma-optical emission spectrometry (ICP-OES; 5800 ICP-OES, Agilent, Santa Clara, CA). To evaluate the total amount of calcium ions loaded, each of the SFBK scaffolds (6-mm diameter/3-mm thickness) was completely dissolved in an acidic solution (1 ml) used by the operator for complete dissolution followed by ICP-OES. To determine the released amount of calcium ions, each of the SFBK scaffolds was placed onto 24-well plates containing 1 ml of PBS/well and incubated at 37 °C for various times (1 to 20 d). PBS was replaced with the same fresh solution per day, and at various time points (1, 5, 10, 15, and 20 d) after incubation, the scaffold-soaked PBS was harvested to determine the released amount of calcium ions. The concentration of the scaffold-released calcium ions was calculated based on the calibration curve obtained from variously diluted calcium solutions (0, 0.1, 0.2, 0.4, 0.6, 0.8, 1, 5, 10, and 20 ppm). The in vitro release profile of calcium ions was also determined by calculating the ratio (%) of the cumulative mass of calcium ions released at the indicated time points to the total amount contained in the SFBK scaffold. Alternatively, the amount of SDF-1 released from the SFBK-S1 scaffold was determined by liquid chromatography and high-mass-accuracy time-of-flight mass spectrometry (LC/TOF-MS). Briefly, each of the scaffold was immersed into 1 ml of PBS and incubated for 6 d. PBS was newly replaced and harvested after an additional 24-h incubation for further analysis. The separation in LC was conducted using an Acquity UPLC system (Waters, Manchester, UK) with an Acquity UPLC Protein BEH C4 column (300 Å, 1.7 μm, 2.1 × 100 mm). All MS data were recorded in positive ion (electrospray ionization) mode, and the data were processed on the Agilent MassHunter Workstation data acquisition software for qualitative analysis. The electrospray-ionization-positive capillary voltage and cone voltage were set at 3.0 kV and 40 V, respectively. The release of SDF-1 from the SFBK-S1 scaffold in vitro was also quantified by enzyme-linked immunosorbent assay (ELISA). To this end, each of the SFBK-S1 scaffolds was initially soaked in a conical tube containing 2 ml of PBS and incubated at 37 °C in a shaking incubator. At various time points (6 h, 12, h, 24 h, 2 d, 3 d, 5 d, 7 d, 10 d, 14 d, and 28 d), PBS was collected and frozen at −80 °C prior to use. An equal volume of fresh PBS was added to the tube immediately after collection at the desired times. The concentration of SDF-1 in the collected samples was determined using a human CXC motif chemokine 12/SDF-1 αELISA kit (Catalog No. DSA00/Lot No. P397191, R&D Systems, Minneapolis, MN, USA) according to the manufacturer’s instructions. The cumulative amount of released SDF-1 was calculated as the ratio (%) of the cumulative mass of SDF-1 released at the indicated times to its initial amount cross-linked per the scaffold.

### Cell proliferation assays

The use of Cell Counting Kit-8 (CCK-8; Dojindo Molecular Tech., Kumamoto, Japan) allowed colorimetric evaluation of the scaffolds’ cell proliferation properties and cytotoxicity. In this study, human mesenchymal stem cells (hMSCs; C-12974, PromoCell GmbH, Heidelberg, Germany) were cultured on SF or SFBK scaffolds attached to 96-well culture plates in growth medium (α-minimum essential medium supplemented with 20% fetal bovine serum [Waltham, MA, USA], 100 IU/ml penicillin G, and 100 μg/ml streptomycin) at 37 °C for 1, 3, and 7 d. At the end of incubation, 60 μl of CCK-8 solution was added into the cultures with an additional 2-h incubation. Thereafter, the CCK-8-containing medium (100 μl) was collected from each well of the plate and transferred to a new 96-well culture plate. The formazan-dye-specific absorbance was determined at 405 nm using a microplate reader (SPECTROstar Nano, BMG LABTECH, Ortenberg, Germany).

### Cell migration assay and morphological analysis

This study evaluated the effect of SFBK with SDF-1 cross-linking on the migration of cells. To this end, a growth medium (100 μl) containing hMSCs (10^4^ cells/ml) was spread on SFBK or SFBK-S1 scaffolds in a 96-well culture plate. After incubation for 12 h, the scaffolds were transferred into a new 96-well plate containing only the culture medium (100 μl) and incubated for an additional 3 d. The scaffolds were fixed in cold 4% paraformaldehyde (PFA) solution and stained with ActinGreen 488 dye (ActinGreen 488 ReadyProbes, Thermo Fisher Scientific) and 4′,6-diamidino-2-phenylindole (DAPI). The stained cells were examined under a confocal laser scanning microscope (Carl Zeiss, Oberkochen, Germany), and the migration distance and length of cells on the scaffolds were analyzed using the ImageJ software with an equal number of randomly selected cells.

### Immunofluorescence assay

A culture suspension (200 μl) containing 10^3^ human umbilical vein endothelial cells (HUVECs; CRL-1730, American Type Culture Collection, Manassas, VA, USA) was spread onto SF, SFBK, or Ang1-conjugated SFBK scaffolds. The cultures of hMSCs were also seeded on SF, SFBK, or SFBK-S1 scaffolds in growth medium. After incubation for 3 d, the scaffolds were fixed in 4% PFA solution and washed 3 times with PBS. Scaffolds were incubated with 0.25% Triton X-100 in PBS for 10 min and washed with PBS containing 0.05% Tween detergent in PBS (PBS-T). Scaffolds were further incubated for 30 min in 50 ml of PBS-T solution containing 0.5 g of bovine serum albumin and 0.375 g of glycine. After washing and blocking processes, scaffolds were exposed to anti-vascular endothelial growth factor (anti-VEGF; 1:250, Abcam, Cambridge, UK), anti-osterix (1:200, Abcam), anti-runt-related transcription factor 2 (anti-RUNX2; 1:250, Santa Cruz Biotechnology, Santa Cruz, USA), or anti-SDF-1 (1:200, Abcam) antibodies. Thereafter, scaffolds were incubated with secondary Alexa Fluor 488-conjugated anti-goat immunoglobulin G (IgG; 1:500, Abcam), Alexa Fluor 594-conjugated anti-rabbit IgG (1:1,000, Abcam), or Alexa Fluor 488-conjugated anti-mouse IgG (1:1,000, Abcam) antibody, followed by counterstaining with aqueous mounting medium (Santa Cruz Biotechnology) containing DAPI at room temperature for 10 min. Finally, the expression patterns of antibody-specific molecules were evaluated by confocal laser scanning microscopy (Carl Zeiss).

### Animals and marking with a noninvasive method

Male Sprague–Dawley (SD) rats (7 weeks old) were purchased from Damul Science (Daejeon, South Korea) and randomly assigned to the experimental groups. Rats were equilibrated for 7 d before the surgery to implant ring-shaped scaffolds for in vivo vascularization or to create calvarial defects. The rats used for calvarial defects were marked by skin staining with nontoxic dyes as a noninvasive approach. During the experimental periods, all animals were housed at 22 ± 1 °C and 55% ± 5% humidity with free access to food and water.

### Implantation of ring-SFBK scaffolds into a rat model for vascularization

To produce VR-SFBK scaffolds, 15 male SD rats (8 weeks old) were injected intramuscularly with Zoletil (0.4 ml/kg, Virbac Laboratories, Carros, France) blended with Rompun (10 mg/kg, Bayer Korea Ltd., Seoul, South Korea). After anesthesia, the femoral arteries and veins were identified through a 1.5-cm incision in the inguinal region and isolated for implantation into the ring-SFBK scaffolds that were conjugated with Ang1 and/or incubated with HUVECs. To induce vascularization in the ring-SFBK scaffolds, an axial blood supply was introduced through a 1.5-mm hole. The inside of the skin was sutured with interrupted sutures using 6-0 chromium absorbent viscera and the outside using 4-0 silk. At 5 d postimplantation with the ring-SFBK scaffolds, the rats were sacrificed. Postmortem perfusion was performed to visualize the vascular networks formed in the scaffolds.

### Puzzle fitting and implantation of scaffolds into calvarial bone defects

The SFBK or SFBK-S1 scaffolds were fitted into the center of ring-SFBK or VR-SFBK scaffolds immediately before implantation into a rat model of calvarial defects. In brief, anesthesia was performed by intramuscular injection of Zoletil mixed with Rompun, and defects 6 mm in diameter were created in the calvarial bones of 30 male SD rats (8 weeks old). The defects were implanted with the puzzle-fitted scaffolds including the SFBK-S1-fitted ring-SFBK without the in vivo vasculogenic process (ring-SFBK [R-SFBK]/SFBK-S1 group), the SFBK-fitted VR-SFBK (VR-SFBK/SFBK group), and the SFBK-S1-fitted VR-SFBK scaffolds (VR-SFBK/SFBK-S1 group). The rats that did not undergo implantation with scaffolds were used as the sham group. Postoperatively, the calvarial defects were sutured with 4-0 silk, and 20 mg/ml amikacin was administered by intramuscular injection at a dose of 0.15 ml/kg. During the experiment, all SD rats were housed at 22 ± 1 °C with free access to food and water. At various times postsurgically, the quantity and quality of newly formed bones at calvarial defects of the sham and experimental groups were evaluated by micro-computed tomography (μCT) and histological analyses.

### μCT analysis

SD rats implanted with the ring-SFBK scaffolds for in vivo vascularization were sacrificed at 5 d postsurgery, and the cadavers underwent perfusion with a radiopaque fluid. Briefly, blood was removed from the specimens by left ventricular intubation and right atrium transection for outflow with warm, PBS-containing heparin. When the peripheral organs blanched, the samples were treated with 4% PFA and perfusion medium of Microfil solution (Flow Tech, Carver, MA, USA). At the end of the procedure, the intratissue Microfil solution was refrigerated for 24 h to harden. The samples underwent additional fixation with 4% PFA, followed by μCT analysis. The formation of new bone at calvarial defects was also determined by live μCT analysis at 4, 8, and 12 weeks after implantation with scaffolds. New bone formation and radiopaque fluid-filled vasculature were visualized using the SkyScan 1076 microfocus x-ray system (SkyScan, Kontich, Belgium) with the CT software CTAn 1.8, CTvol, and NRecon Reconstruction (SkyScan).

### Histological analyses

The ring-scaffolds that were implanted around the rats’ femoral vessels were collected and fixed in 4% PFA at 5 d postimplantation. After xylene treatment and rehydration, samples were paraffin-embedded, sectioned (9 μm in thickness), and subjected to hematoxylin and eosin (H&E) as described previously [19]. The aspect of newly formed blood vessels in the implanted scaffolds was observed in relation to the loading of Ang1, HUVECs, or both. Calvarial bones including the defect regions were also subjected to H&E, Masson’s trichrome, or immunohistochemical (IHC) staining as described previously [19]. In brief, the bone samples were isolated at 12 weeks after scaffold implantation and decalcified for 8 weeks in 17% EDTA at 4 °C after fixation with a 4% PFA solution for 48 h. Samples were dehydrated, embedded in paraffin, and serially and perpendicularly sectioned (9 μm in thickness) prior to staining. In the IHC assay, anti-osterix, anti-osteocalcin (anti-OCN), and anti-tumor necrosis factor-α (anti-TNF-α) antibodies were applied at 1:200 dilution and their expression patterns were visualized using an IHC accessory kit (PK-6101, Vector Laboratories, Burlingame, CA, USA) according to the manufacturers’ instructions. The stained tissue samples were observed and photographed using MoticEasyScan One and Motic DSAssistant (Kowloon, Hong Kong).

### Statistical analyses

All data are presented as mean ± standard deviation. Differences between 2 groups were analyzed by an unpaired Student *t* test with parametric test and Welch’s correction using the GraphPad Prism (ver. 9.5) program. One-way analysis of variance (ANOVA) followed by Tukey’s multiple-comparisons test was used for multiple comparisons among more than 2 groups using the same program. A *P* value <0.05 was considered statistically significant.

## Results and Discussion

### A novel strategy to enhance synergistic bone regeneration via the puzzle-fitting process of bioengineered and vascularized SFBK scaffolds

**Fig. 1.**
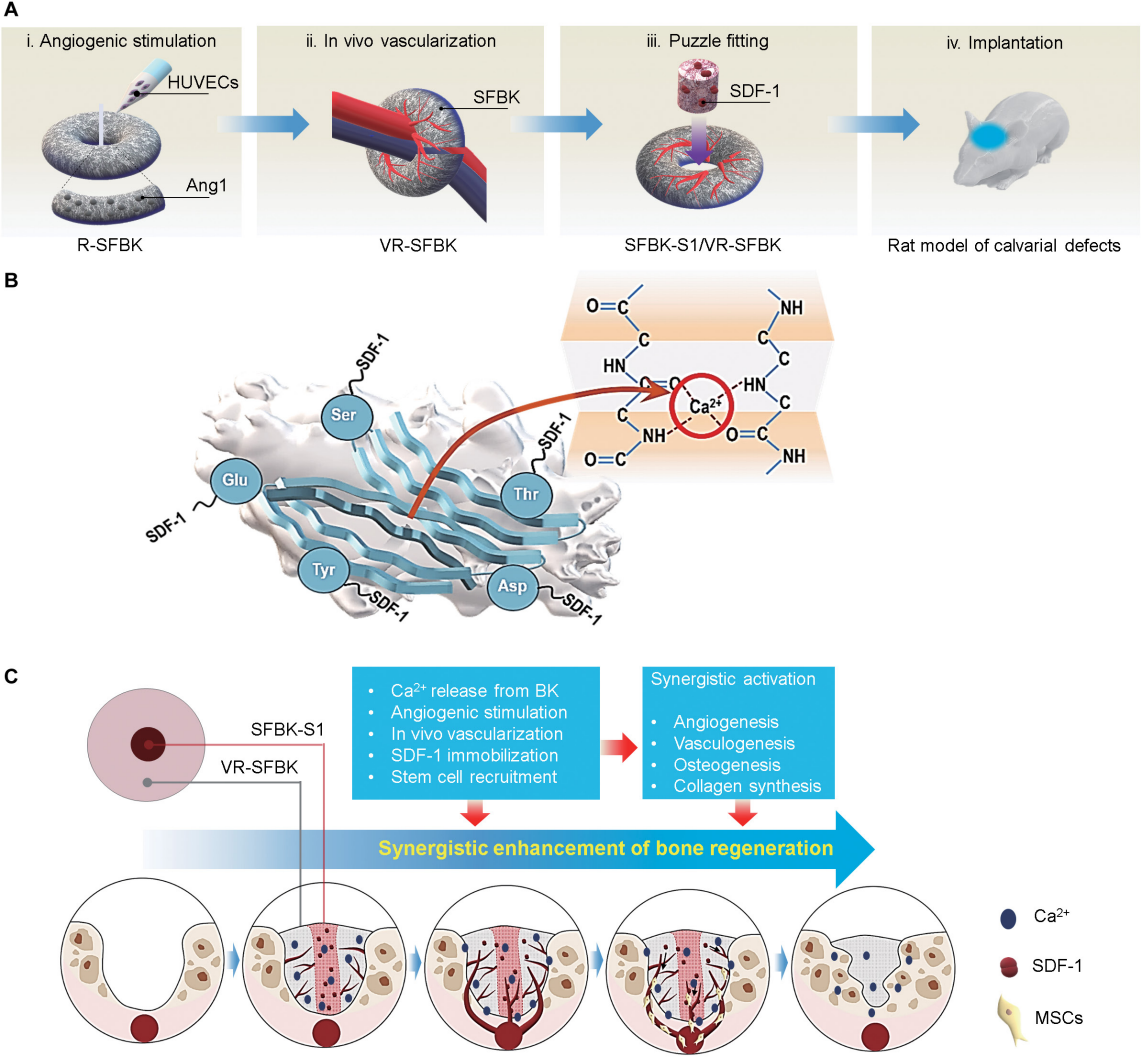
Schematic illustrations showing the mechanisms by which the puzzle-fitted silk fibroin-mixed *Broussonetia kazinoki* (SFBK) scaffolds synergistically enhance bone regeneration in a rat model of calvarial defects. (A) Illustrations exhibiting the procedures for angiogenic stimulation, vascularization, puzzle fitting, and implantation of SFBK scaffolds. (B) Schematic diagram of silk fibroin (SF) molecular chains in reconstituted SFBK with calcium ions interfering with hydrogen bonding and the secondary structure after 1-ethyl-3-(3-dimethylaminopropyl)carbodiimide (EDC)/*N*-hydroxysuccinimide (NHS) activation. (C) The mechanisms by which the puzzle-fitted SFBK scaffolds enhance bone regeneration. HUVECs, human umbilical vein endothelial cells; Ang1, angiopoietin-1; R-SFBK, ring-shaped SFBK; VR-SFBK, vascularized ring-shaped SFBK; SDF-1, stromal cell-derived factor 1; SFBK-1, SDF-1-cross-linked scaffold; BK, *B. kazinoki*; MSCs, mesenchymal stem cells.

The experimental designs aimed to regenerate large bone defects through the puzzle-fitting process and the associated mechanisms by which the puzzle-fitted scaffolds enhance bone healing are illustrated in Fig. 1. Figure 1A represents novel approaches to producing Ang1-linked and HUVEC-cultured SFBK scaffolds, to yielding VR-SFBK scaffolds, to combining VR-SFBK with SFBK-S1 scaffolds via the puzzle-fitting process, and to implanting the puzzle-fitted scaffolds into a rat model of calvarial defects. The main points of these approaches might be the combined application of angiogenic and chemoattracting biomolecules such as Ang1 and SDF-1 into calcium-releasing scaffolds and the puzzle-fitting process for their synergistic interactions. Numerous studies have indicated that the vasculogenic or stem-cell-recruiting strategy is useful for efficient and successful healing of defect bones, and this efficacy is further enhanced by its application in a combination [6,7,11–13,20]. Figure 1B indicates the linkage of SDF-1 with SFBK scaffolds through a direct reaction with the primary amine of the protein after the EDC/NHS activation process together with the calcium-ion-mediated bonding. A silk-based scaffold is an effective cross-linking material that reacts with amino acids or proteins containing residues with primary amine groups in the EDC/NHS activation process [21,22]. As the direct delivery of SDF-1 to tissues can cause its rapid diffusion and degradation by proteolytic enzymes, many researchers have tried to use biomaterials that control the delivery and degradation of the chemokine [20]. To cross-link SDF-1 to silk-based scaffolds, the EDC/NHS process is also widely applied because of its potencies in conjugating and applicating of bone tissue regeneration [23,24]. Taken together, Fig. 1C illustrates a schematic demonstration of our hypothesis that the puzzle-fitted scaffolds promote bone regeneration at defects via the synergistic activation of angiogenesis, vasculogenesis, collagen synthesis, and osteogenesis. We also hypothesize that this synergistic activation involves the release of calcium ions from BK, angiogenic stimulation by treatment with HUVECs and Ang1, in vivo vascularization, and SDF-1-induced stem cell recruitment.

### Mechanism of the effect of calcium ions on the structure of scaffolds

**Fig. 2.**
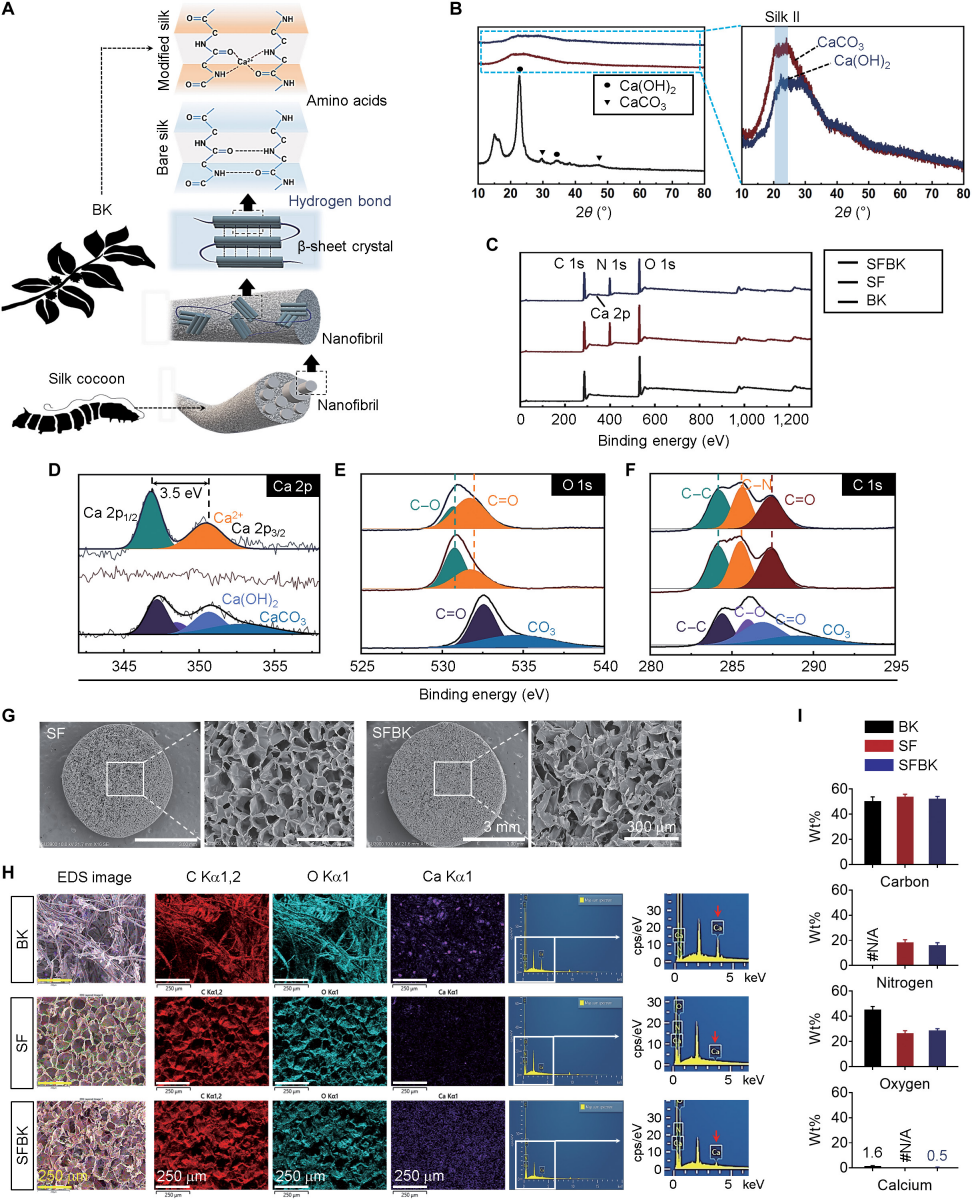
Physiochemical characterization of the effect of calcium ions on the SF structure in an SFBK scaffold. (A) Schematic diagram of calcium ions extracted from BK entrapped in crystallized silk. Calcium ions entrapped in silk crystals can be released relatively slowly from the body, creating a stable mixture in water. (B) X-ray diffraction (XRD) graphs of BK, SF, and SFBK scaffold loading with different calcium hydroxide and calcium carbonate ratios. (C) X-ray photoelectron spectroscopy (XPS) survey spectra of BK, SF, and SFBK scaffolds along with the corresponding high-resolution XPS survey spectra of (D) Ca 2p, (E) O 1s, and (F) C 1s. (G) Scanning electron microscopy (SEM) images of SF and SFBK scaffolds. (H) Energy-dispersive spectroscopy (EDS) analysis and (I) quantitative graph showing the calcium content in BK, SF, and SFBK scaffolds (*n* = 5). #N/A, not available.

A mechanism by which BK-derived calcium ions affect the secondary structure of SFBK scaffolds is proposed in Fig. 2A. While BK was boiled in 0.1 M NaOH-supplemented distilled water to remove nonfiber materials, BK-derived CaCO_3_ reacted with NaOH to produce CaOH_2_. The CaOH_2_ contained in the BK powder dissolved in the silk mixture solution to release calcium ions that were chelated with silk chains to form a cross-linked network. Figure S1 shows the digital (a and b) and SEM images (c and d) of BK, SF, and SFBK scaffolds. Based on the SEM images, SFBK, which contains calcium ions that chelate with silk chains to form a cross-linked network, has a porosity similar to that of SF despite having slightly smaller pores (Fig. S1d). The formation of a cross-linked network by chelation of calcium ions in SFBK scaffolds into silk chains was confirmed by XRD and XPS analyses. BK-containing SF samples exhibited the typical XRD diffraction peaks at 22.0° that correspond to a typical silk crystal structure (Fig. 2B, left panel). After blending with BK, the silk crystal’s main peak at 22.0° remained obvious in SFBK but with a decreased intensity. An additional peak representing CaCO_3_ was also observed at 30.0° (Fig. 2B, right panel). With BK, the intensity of silk crystal peaks decreased and presented as an amorphous structure, indicating that calcium ions prevent silk crystallization. The XPS results in Fig. 2C to F further demonstrate the presence of CaOH_2_ and CaCO_3_ in BK and chelation of calcium ions in silk crystallization in SFBK. Typical XPS wide-scan spectra from BK, SF, and SFBK are presented in Fig. 2C. All 3 elements comprising CaCO_3_ and CaOH_2_, i.e., calcium, oxygen, and carbon, were observed. High-resolution XPS spectra of the Ca (2p) core levels of BK and SFBK were confirmed, and the chemical shifts between the Ca 2p_3/2_ and Ca 2p_1/2_ core levels in both samples were approximately 3.5 eV (Fig. 2D). O 1s and C 1s XPS high-resolution spectral results are typically used to identify functional group changes in modified silk layers. The maintenance of carbon and oxygen contents in SFBK was comparable to that in SK, and the C–C/C–N/C=O components in SFBK were also like those in SF without a prominent increase or decrease (Fig. 2E and F). In the lyophilization process, SF alone or powdery BK-mixed solution was converted into porous SFBK scaffolds using ice crystals as a template. The volatilization of the ice crystals contributed to the formation of many homogeneous 3-dimensional macropores larger than 50 μm and smaller than 100 μm within both SF and SFBK (Fig. 2G). In addition, the chemical compositions of BK, SF, and SFBK were investigated by displaying the EDS spectrum as an elemental distribution color map (Fig. 2H and I). The EDS color map and spectrum demonstrate that carbon was the main element among all BK, SF, and SFBK. Oxygen and nitrogen were also present. The homogeneous distributions of carbon and nitrogen were identical in the color maps, with no significant differences between the scaffolds. However, calcium, which appeared prominently in the EDS analysis of BK, was found irregularly on the fiber surface, was absent from SF, and was evenly distributed throughout SFBK (Fig. 2H). The EDS analysis also confirmed the presence of calcium at average concentrations of 1.6 and 0.5 wt% inside BK and SFBK, respectively (Fig. 2I). The concentration of SF may affect silk crystallization and calcium ion chelation. From the perspective of the biological function of calcium ions in bone regeneration, we suggest that calcium ions contained in BK are appropriately released and promote the osteogenic differentiation and migration of mesenchymal stem cells [25,26]. Therefore, we consider that the combination of SF, a biocompatible structural protein, with BK, a source of calcium ions, might improve a poorly adhesive surface for cells and increase the bioactivity and protein adsorption that are required for successful tissue engineering [17].

### Design of SFBK scaffolds for the puzzle-fitting process, mechanical and degradation properties of scaffolds, and their releasing patterns of calcium ions and SDF-1

**Fig. 3.**
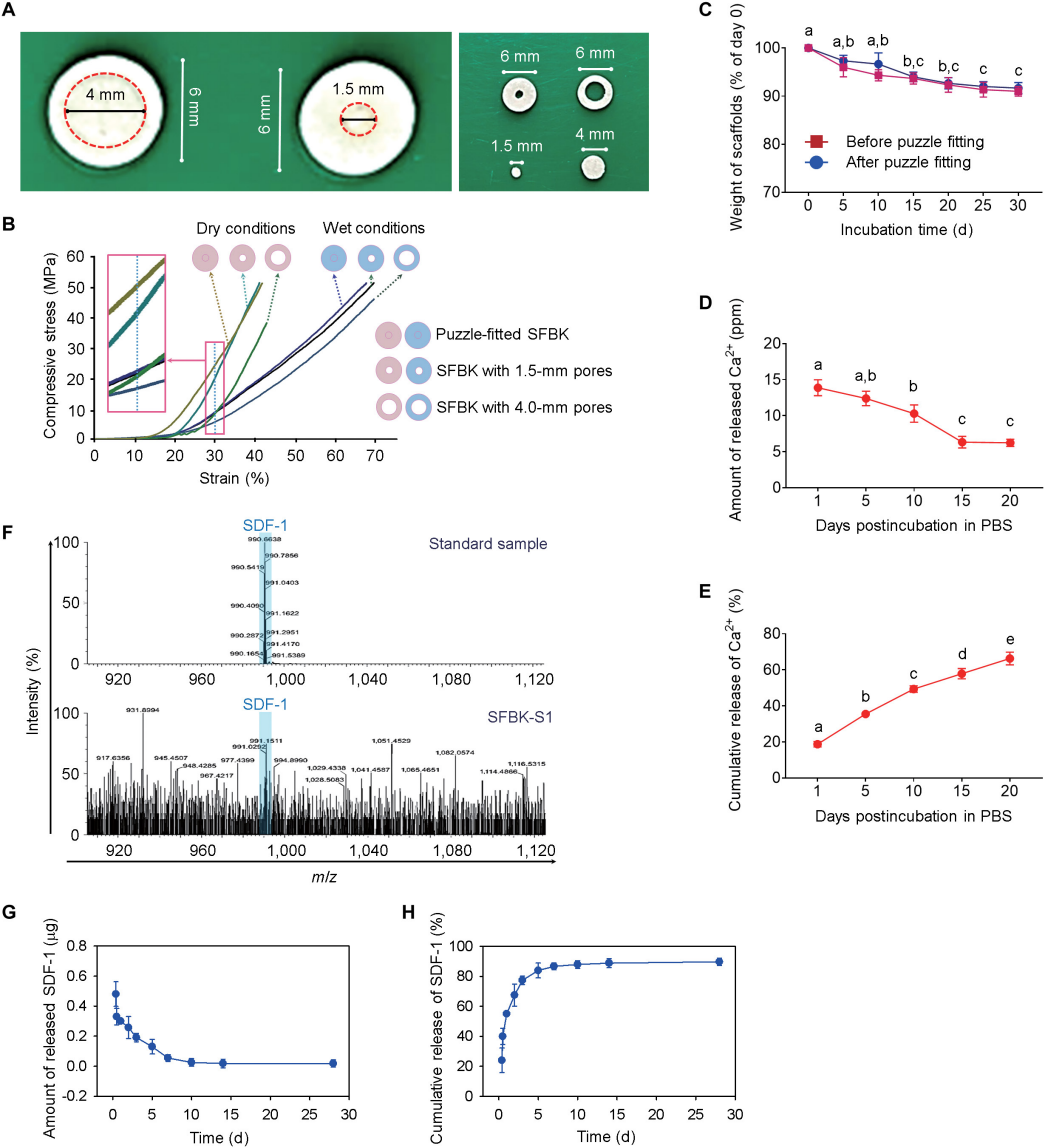
Construction of the designed SFBK scaffolds, determination of mechanical and degradable properties, and releasing patterns of calcium ions and SDF-1. (A) Digital photos of ring-SFBK scaffolds before and after the creation of a hole in the center. (B) Compression stress–strain curves of the designed SFBK scaffolds in wet and dry conditions before and after the puzzle-fitting process. (C) Degradable properties of SFBK scaffolds before and after the puzzle-fitting process, in which the weight changes in the scaffolds were measured at the indicated days after incubation in phosphate-buffered saline (PBS) and expressed as % of their initial weights. (D) The time-dependent pattern of SFBK scaffolds to release calcium ions at the indicated time points of incubation in PBS. (E) Cumulative release (%) of calcium ions from SFBK scaffolds at the indicated days after incubation in PBS. (F) Total ion chromatogram (TIC) of full time-of-flight mass spectrometry (TOF MS) scan of the standard and SFBK-S1 scaffolds indicating the presence of a peak corresponding to SDF-1 at 990 *m*/*z*. (G) The amount of SFBK-S1-scaffold-released SDF-1 at the desired time points of incubation (*n* = 3). (H) Cumulative release (%) of SDF-1 from the scaffolds at the indicated times after incubation. The letters a to e indicate significant differences (*P* < 0.05) among the groups from ANOVA followed by Tukey’s multiple-comparisons test (*n* = 3).

A central 1.5- or 4-mm hole was created in SFBK scaffolds (6-mm diameter) to yield ring-shaped and round scaffolds for in vivo vascularization and cross-linking with SDF-1, respectively. Figure 3A shows photographs of SFBK scaffolds before (left panel) and after creation of a hole in the center of the scaffolds (right panel). We initially determined the mechanical strengths of the designed scaffolds under dry and wet conditions. Scaffolds in wet conditions expressed a higher strain (%) than did those in dry conditions regardless of the central pore creation or the puzzle fitting (Fig. 3B). When the compressive stresses (MPa) of the differently designed scaffolds were compared at a strain of 30%, the puzzle-fitted SFBK with a 1.5-mm round scaffold showed the highest strength in both dry and wet conditions, whereas the ring-shaped scaffold with a 4-mm central pore revealed the lowest strength in the same conditions. However, the ring-shaped scaffold puzzle-fitted with a 4-mm round scaffold exhibited a strength similar to that with a 1.5-mm round scaffold in dry and wet conditions (data not shown). These results indicate that the mechanical strength of ring-shaped scaffolds is not directly affected by the size of the pore created in the center under the condition of puzzle fitting with round scaffolds. A fundamental property that needs to be considered when designing a bone scaffold is its degradation rate. Scaffolds should simultaneously be stable enough to allow native cells to populate their surface/bulk and degrade at a suitable speed for the controlled release of bioactive molecules or subsequent native tissue regeneration. We conducted degradation tests on SFBK scaffolds that received puzzle fitting or not by measuring changes in weights before and after incubation in PBS for various days. The results showed that the scaffolds underwent continuous degradation during the incubation regardless of the puzzle fitting (Fig. 3C). At the end of incubation (30 d postimmersion in PBS), both the scaffolds revealed an approximately 10% weight loss compared to their initial dry weights. This result indicates the long-term maintenance of the scaffolds after their in vivo implantation followed by slow degradation. We found that each of the SFBK scaffolds (6-mm diameter) without a hole created contained approximately 74 ppm of calcium ions through the ICP-OES analysis. That analysis also revealed the time-dependent release of calcium ions from SFBK scaffolds, in which the average amount of calcium ions released per scaffold was 13.9 ± 1.1 ppm on day 1, decreased by 6.3 ± 0.5 ppm on day 15, and maintained until day 20 (Fig. 3D). When the cumulative release (%) of calcium ions was calculated, the initial burst release was 18.7% of the total amount (74 ppm/SFBK scaffold) at the first day, and such release was maintained for the experimental periods (Fig. 3E). At 20 d of immersion in PBS, approximately 66% of calcium ions were released from the SFBK scaffold. Calcium is an important signaling messenger to various cellular events involved in proliferation and osteogenic activation for bone regeneration [27,28]. Accordingly, this result indicates that in addition to the degradation property of SFBK scaffolds, their continuous and long-term releasing pattern of calcium ions contributes to a prolonged activation of calcium-ion-stimulated osteogenic response.

In contrast, local application of SDF-1α stimulates the efficacy of multipotent stem cell transplantation in therapeutic bone healing [29]. The released amount of SDF-1 in round scaffolds and the formation of in vivo blood vessels in ring-shaped scaffolds could be controlled by adjusting the internal scaffold diameter. In this study, we evaluated the releasing pattern in vitro of SDF-1 from the SFBK-S1 scaffold via LC/TOF-MS and ELISA. Figure 3F shows the deconvoluted mass spectra of the standard and SFBK-S1 samples at 7 d postimmersion in PBS and indicates the presence of SDF-1 having a molecular weight of around 10 kDa at 990 *m*/*z* in the SFBK-S1-derived fraction. In addition, we did not find a loss of SDF-1 from the SFBK-S1 scaffolds during the detoxifying process with distilled water (data not shown). This indicates that SFBK-S1 scaffolds contained the initially cross-linked amount (2 μg/scaffold) of SDF-1. The results from ELISA revealed a relatively rapid release of SDF-1 from the scaffolds in a time-dependent manner up to 7 d of incubation along with its sustained and continued release with a very small amount until 28 d of incubation (Fig. 3G). When the cumulative amount of released SDF-1 was calculated, 55% of the total SDF-1 was released at 24 h, and nearly 87% release of SDF-1 was observed at 7 d of incubation (Fig. 3H). A considerable study reported a relatively rapid release of SDF-1 regardless of the types of SF-based composite scaffolds, in which approximately 90% of SDF-1 was released from SF scaffolds within 10 d of incubation [30]. Similarly, our results indicate that SDF-1 might be released from SFBK scaffolds more acutely and largely than calcium ions, although the chemokine seems to be released for as long as 28 d with a very small amount. However, we could not exclude the possibility that chemical conjugation of SDF-1 to the scaffold was not successful. This is because the release of SDF-1 from the scaffold is much faster than the degradation rate of the scaffold, although chemical cross-linking of the protein to the scaffold was applied. Taken together, we selected round scaffolds with a 1.5-mm diameter for cross-linking with SDF-1 before puzzle fitting into ring-shaped SFBK scaffolds. Furthermore, all in vivo and in vitro experiments were performed using the SFBK-1 scaffolds (1.5-mm diameter) cross-linked with 2 μg/scaffold and the SFBK scaffolds (6.0-mm diameter) containing 74 ppm/scaffold of calcium ions.

### In vivo vascularization strategy for ring-SFBK scaffolds

**Fig. 4.**
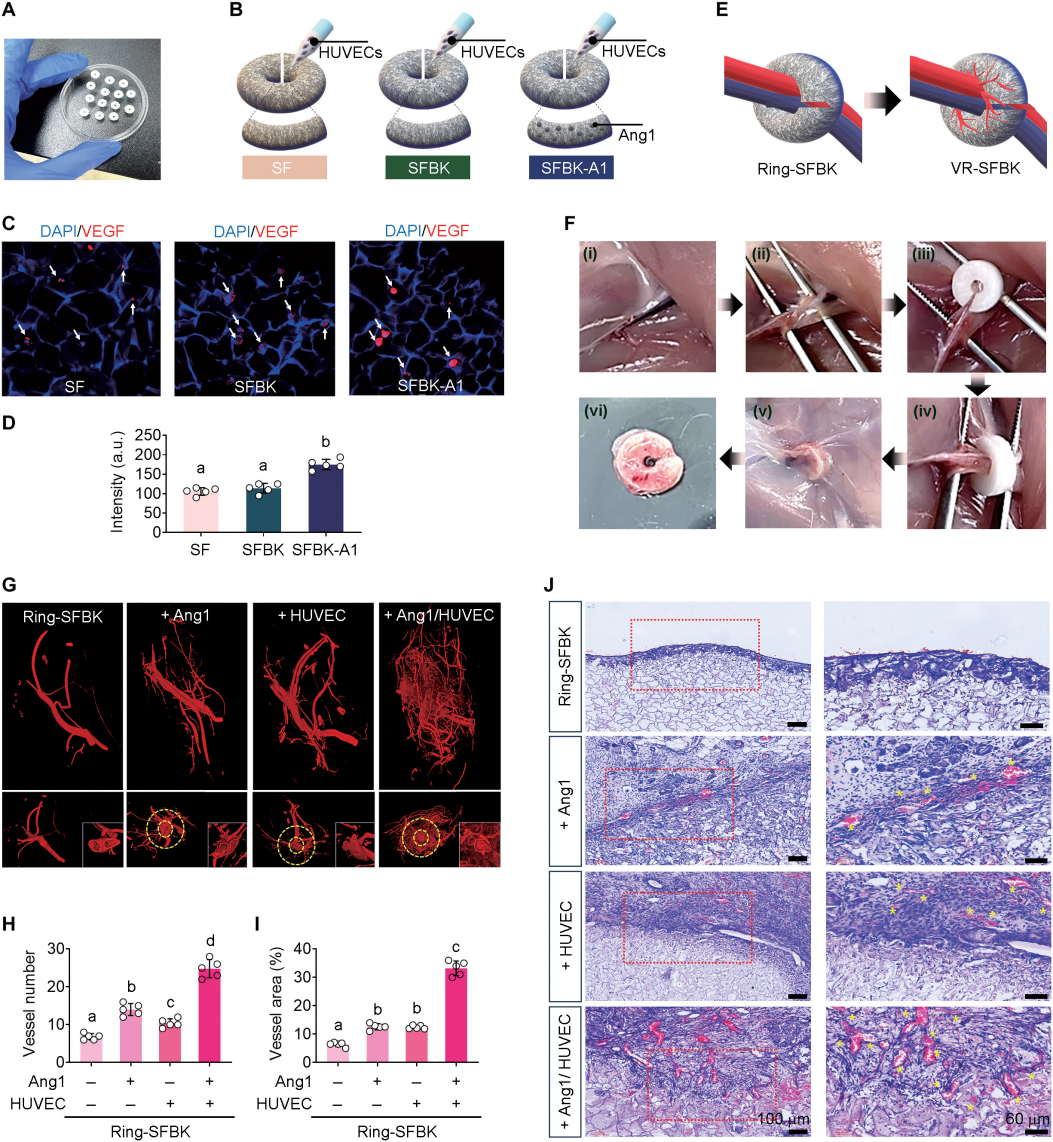
The strategy to develop VR-SFBK scaffolds and their vasculogenic efficacies in relation to HUVEC culture, Ang1 conjugation, or both. (A) Photograph showing the ring-SFBK scaffolds with a hole (1.5-mm diameter) in the center. (B) Illustrations showing angiogenic stimulation of SF and SFBK scaffolds via HUVEC culture, Ang1 loading, or both. (C) Vascular endothelial growth factor (VEGF)-specific immunofluorescence of HUVECs cultured on an SF, SFBK, or SFBK-A1 scaffold at 5 d postincubation along with (D) the quantitative intensities (a.u.). (E) Illustrations indicating the strategy to produce VR-SFBK scaffolds by assembling peripheral blood vessels into the center of ring-SFBK scaffolds. (F) Photographs showing the surgical procedures to induce in vivo vascularization in ring-SFBK scaffolds along with the actual image of an isolated VR-SFBK scaffold at 5 d postimplantation. (G) The micro-computed tomography (μCT) images showing blood vessels formed in the implanted ring-SFBK scaffolds in relation to Ang1 conjugation, HUVEC culture, or both. The (H) numbers and (I) areas (%) of newly formed blood vessels are determined based on the angiographic images. (J) Hematoxylin and eosin (H&E) staining images exhibiting the formation of new microvessels in the indicated scaffolds at 5 d postimplantation. The letters a to d indicate significant differences (*P* < 0.05) among the groups from ANOVA followed by Tukey’s multiple-comparisons test (*n* = 5). DAPI, 4′,6-diamidino-2-phenylindole.

The in vivo angiogenic ability of ring-SFBK scaffolds in relation to HUVEC culture and/or Ang1 immobilization was analyzed to prepare the optimized scaffolds for vascularized and bioengineered bone healing. Figure 4A shows a photograph of ring-SFBK scaffolds with a 1.5-mm hole in the center. Figure 4B shows images of ring-SF or ring-SFBK scaffolds that were cultured with HUVECs, loaded with Ang1, or both. When HUVECs were seeded and incubated on SF, SFBK, and Ang1-immersed SFBK (SFBK-Al) scaffolds for 5 d, the numbers of cells expressing VEGF (white arrows) were comparable among the scaffolds (Fig. 4C). However, the immunofluorescence intensity (a.u.) specific to VEGF in the cells grown on the SFBK-A1 scaffold was markedly higher than that in the cells on the SF or SFBK scaffold (Fig. 4C and D). These results indicate that the Ang1-loaded SFBK scaffold stimulates greater expression of angiogenic factors than the SF or SFBK scaffold alone. As scaffold-mediated healing of large bone defects often requires the formation of microvascular networks within the scaffolds [31], we developed a strategy to produce VR-SFBK scaffolds by implanting ring-SFBK scaffolds into a rat model. Figure 4E illustrates the images of ring-SFBK fitted with blood vessels and the microvascular-network-formed VR-SFBK after in vivo implantation. The photographs in Fig. 4F and Video S1 show the surgical procedures to induce vascularization in ring-SFBK scaffolds along with an image of isolated VR-SFBK. In this study, the processes for in vivo vascularization strategy were (i) retraction of the skin in the abdominal cavity with hooks to expose the femoral sheath after a small skin incision in the inguinal region, (ii) separation of connective tissue surrounding the femoral artery and vein, and (iii and iv) insertion of ring-SFBK scaffolds to enclose the vessels before suturing (Fig. 4F). Figure 4F (v) and (vi) respectively show the VR-SFBK scaffold before and after surgical isolation from the rat model at 5 d postimplantation. To analyze the in vivo angiogenic ability of ring-SFBK scaffolds in relation to treatment with HUVECs, Ang1, or both, vascular structures in the VR-SFBK scaffolds were visualized using a radiopaque fluid. Compared with the ring-SFBK scaffold only, broader formation of blood vessels was found in the scaffolds that received HUVEC culture, Ang1 cross-linking, or both (Fig. 4G). When the number and area (%) of newly formed blood vessels were evaluated, the ring-SFBK scaffolds with HUVEC culture or Ang1 immobilization showed markedly greater increases than the ring-scaffold alone. These increases were further enhanced in the scaffolds treated with both HUVEC and Ang1 (Fig. 4H and I). Like the results from μCT analysis, H&E staining of the histological sections of VR-SFBK scaffolds exhibited greater formation of newly formed blood vessels in HUVEC-cultured or Ang-conjugated scaffolds than in ring-SFBK alone. Synergistic enhancement with the 2 processes was also demonstrated histologically (Fig. 4J). These results indicate that the in vivo vascularization strategy allows a successful neovascularization in the VR-SFBK scaffolds and that strategy involves an orchestrated stimulation of angiogenesis and vasculogenesis [31]. As the Ang1/Ang1 receptor (Tie2) signaling axis tightly regulates vascular development even at the adult stage, the current findings also support the role of supplemental Ang1 to locally and synergistically enhance the healing of bone defects [5,32,33].

### Recruitment of stem cells and the osteo-inductive performance of scaffolds by puzzle fitting SFBK-S1 to VR-SFBK scaffolds

**Fig. 5.**
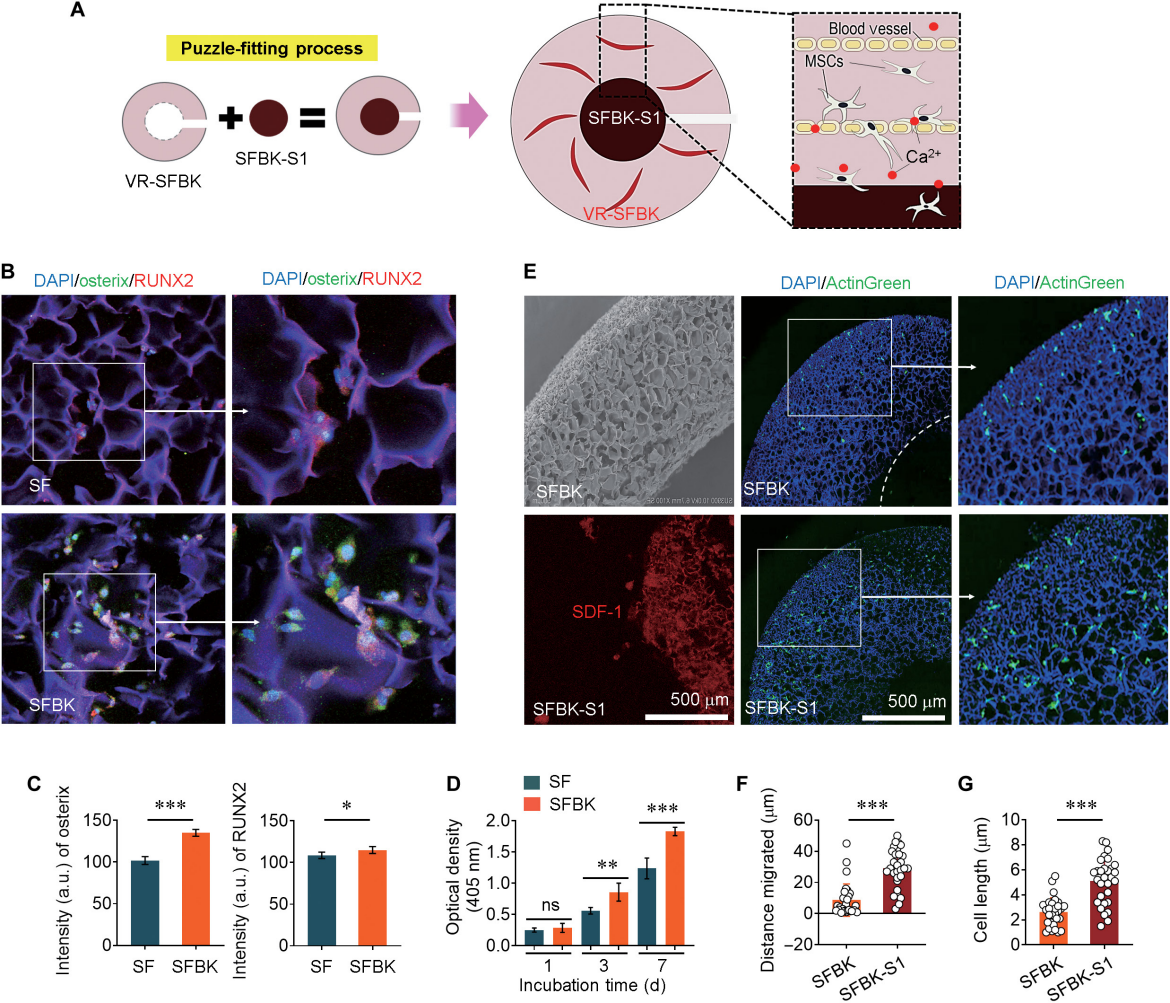
Osteogenic transcription factor expression and migration of human mesenchymal stem cells (hMSCs) on the puzzle-fitted scaffolds in relation to SDF-1 immobilization. (A) Illustration showing the puzzle-fitting process of SFBK scaffolds and the roles of BK-derived calcium ions and cross-linked SDF-1 in osteogenic activation and stem cell recruitment. (B) Confocal laser scanning microscopy (CLSM) images showing osterix- and/or runt-related transcription factor 2 (RUNX2)-specific immunofluorescence in hMSCs cultured for 5 d on an SF or SFBK scaffold. (C) The quantitative intensities (a.u.) of osterix and RUNX2 were determined by the ImageJ software. (D) Proliferation rates of hMSCs grown on SF and SFBK scaffolds were compared by Cell Counting Kit-8 (CCK-8) assay after the indicated incubation period (d). (E) SEM and CLSM images showing the morphology and SDF-1-specific fluorescence of SFBK and SFBK-S1 scaffolds, respectively, along with the SDF-1-stimulated migration of hMSCs. (F) The distances of cell migration and (G) cell length were determined by randomly selecting more than 10 cells/image. *P < 0.05, **P < 0.01, and ***P < 0.001 by unpaired Student *t* test with parametric and Welch’s correction. ns, not significant.

The induction of stem cell recruitment into implanted scaffolds facilitates the healing process in bone defects, and this induction can be accomplished by linking SDF-1 into scaffolds. We designed a strategy that assembles an SDF-1-cross-linked scaffold into the center of a VR-SFBK scaffold via a puzzle-fitting process. This process is intended to stimulate the recruitment of mesenchymal multipotent stem cells toward the center of the scaffolds, accelerating bone regeneration in synergistic combination with VR-SFBK-mediated vascularization and BK-derived calcium release as illustrated in Fig. 5A. In this study, we utilized 1.5-mm round and 6-mm ring-shaped scaffolds for SDF-1 immobilization and in vivo vascularization, respectively. As calcium ions play important roles in bone homeostatic maintenance, we evaluated the effect of the combination of SF and BK on the expression of bone-specific markers in hMSCs. Compared with the cells grown on SF scaffolds, the cells incubated on the SFBK scaffolds had greater expression of osterix, RUNX2, or both (Fig. 5B and Fig. S2). When the immunofluorescence intensities specific to these markers were quantified, the intensities (a.u.) were higher on the SFBK scaffolds than on the SF scaffolds (Fig. 5C). RUNX2 and osterix are transcription factors involved in upregulating the osteoblastic differentiation pathway. RUNX2 is essential for skeletal development by modulating osteoblastic differentiation and chondrocyte growth and maturation [34]. Osterix is required for bone mineralization and the differentiation of preosteoblasts into mature and functional osteoblasts [35]. Accordingly, our results indicate that combination with BK improves the osteo-inductive performance of SF by stimulating the osteogenic molecules of mesenchymal multipotent stem cells. CCK-8 assay results also support that the SFBK scaffold better stimulates the incubation-time-dependent increase in the proliferation of hMSCs than the SF scaffold (Fig. 5D). This result also indicates the noncytotoxic effect of SFBK scaffolds on cells. We further analyzed the effect of puzzle-fitted SFBK on the migration of hMSCs in relation to cross-linking with SDF-1. The number of actin-expressing cells (Fig. 5E), cell migration into the center of the scaffold (Fig. 5F), and cell length (Fig. 5G) were greater in cells grown on SFBK-S1-fitted scaffolds than in cells grown on the SDF-1-free scaffolds. Moreover, the SDF-1-linked SF scaffold augmented the cell length expansion and migration of hMSCs to the same levels as did the SFBK-S1 scaffold (Fig. S3). These results suggest that cross-linking with SDF-1 rather than the composition of scaffolds is a further important factor in inducing the migration of multipotent stem cells into the center of scaffolds. This also indicates that the cross-linking with SDF-1 may allow balanced and uniform formation of new bones in implanted scaffolds.

### In vivo bone repair performance of puzzle-fitted SFBK scaffolds

**Fig. 6.**
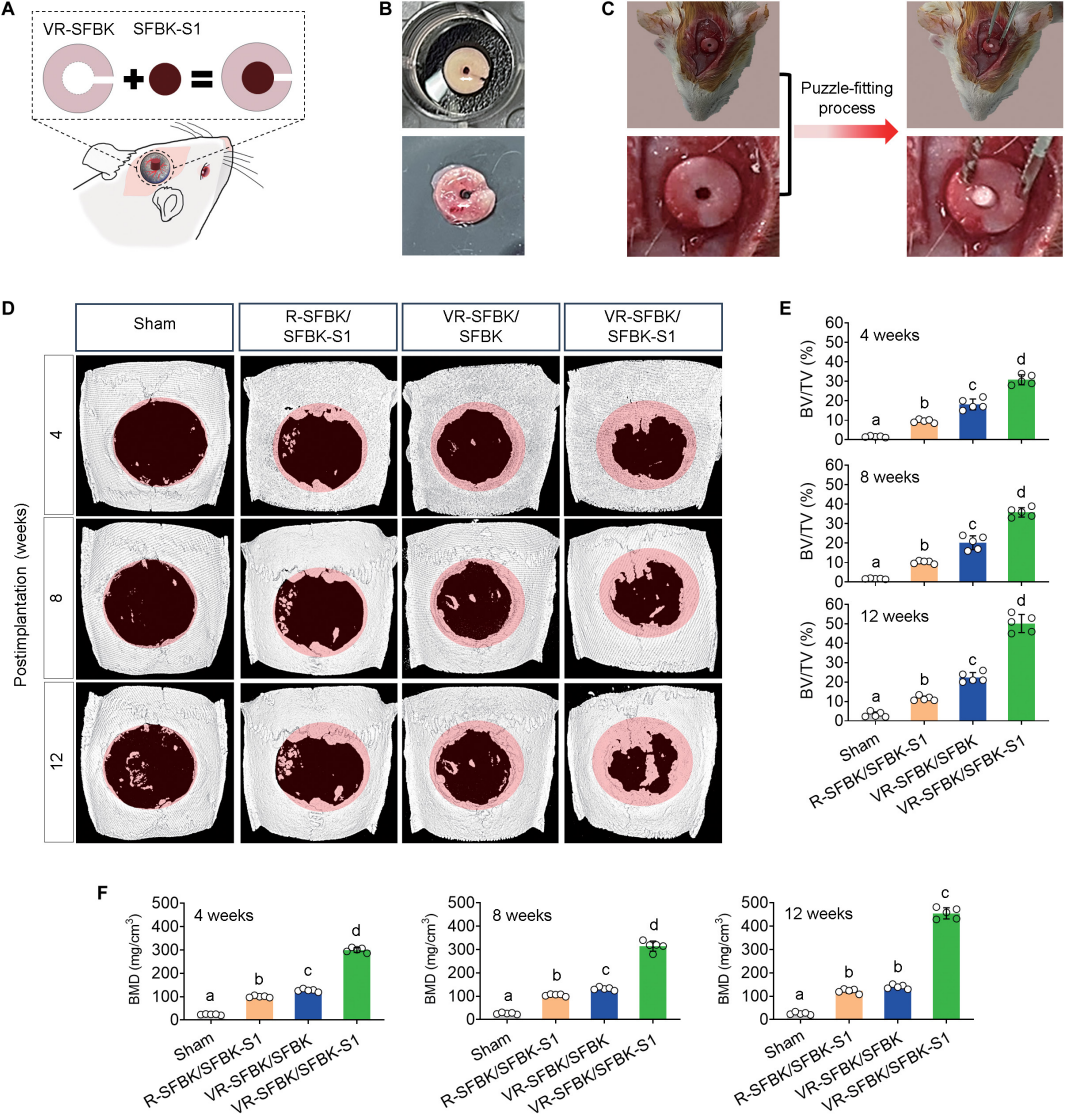
Bone healing efficacies of the puzzle-fitted scaffolds in relation to in vivo vascularization and/or SDF-1 immobilization processes. (A) Illustration showing the implantation of a puzzle-fitted scaffold into a rat calvarial defect model. (B) Digital images of ring-SFBK scaffolds before and after in vivo vascularization. (C) Digital photos showing the puzzle-fitting process of scaffolds in the defect. (D) The μCT images showing calvarial bones including defect regions of the experimental groups along with the values of (E) bone volume over total volume (BV/TV) (%) and (F) bone mineral density (BMD) (mg/cm^3^) at 4, 8, and 12 weeks postimplantation. The letters a to d indicate significant differences (*P* < 0.05) among the groups from ANOVA followed by Tukey’s multiple-comparisons test (*n* = 5).

We investigated the bone healing efficacy of puzzle-fitted SFBK scaffolds in relation to in vivo vascularization, SDF-1 cross-linking, or both using a rat model of calvarial defects. Figure 6A illustrates the puzzle-fitting process of SFBK scaffolds and their implantation into defects. Figure 6B shows photographs of ring-SFBK scaffolds before (upper panel) and after in vivo vascularization (lower panel). Figure 6C displays the in vivo puzzle-fitting process in the rat model of calvarial defects in which the defects were (VR-SFBK) or were not (R-SFBK) first implanted with a ring-SFBK that had undergone in vivo vascularization immediately before fitting. The levels of newly formed bones at defective regions were evaluated by live μCT at 4, 8, and 12 weeks postimplantation. The sham group that received a surgical defect showed only minimal new bone formation at the defect throughout the postsurgical period, with a small quantity of new bone appearing at 12 weeks postsurgery (Fig. 6D). Compared with the sham group, all of the groups implanted with the puzzle-fitted SFBK scaffolds exhibited increased formation of new bone. Among the SFBK-implanted groups, the VR-SFBK/SFBK-S1 group revealed the greatest time-dependent formation of new bones after implantation. The increase in bone regeneration by the SDF-1 cross-linking and/or vasculogenic processes and the enhancement by combination of these processes were supported by the values of bone volume over total volume (BV/TV) (%) and bone mineral density (BMD; mg/cm^3^) (Fig. 6E and F). Specifically, the VR-SFBK/SFBK-1 group showed substantial enhancement in the BMD value compared with the R-SFBK/SFBK-S1 and VR-SFBK/SFBK groups. The implantation with SFBK scaffold alone (6-mm diameter/non-pore creation) also stimulated the healing of bone defect along with substantial increases in the BV/TV and BMD values compared with those of sham group (Fig. S4). However, these increases were inferior to those by R-SFBK/SFBK-S1 and VR-SFBK/SFBK groups.

**Fig. 7.**
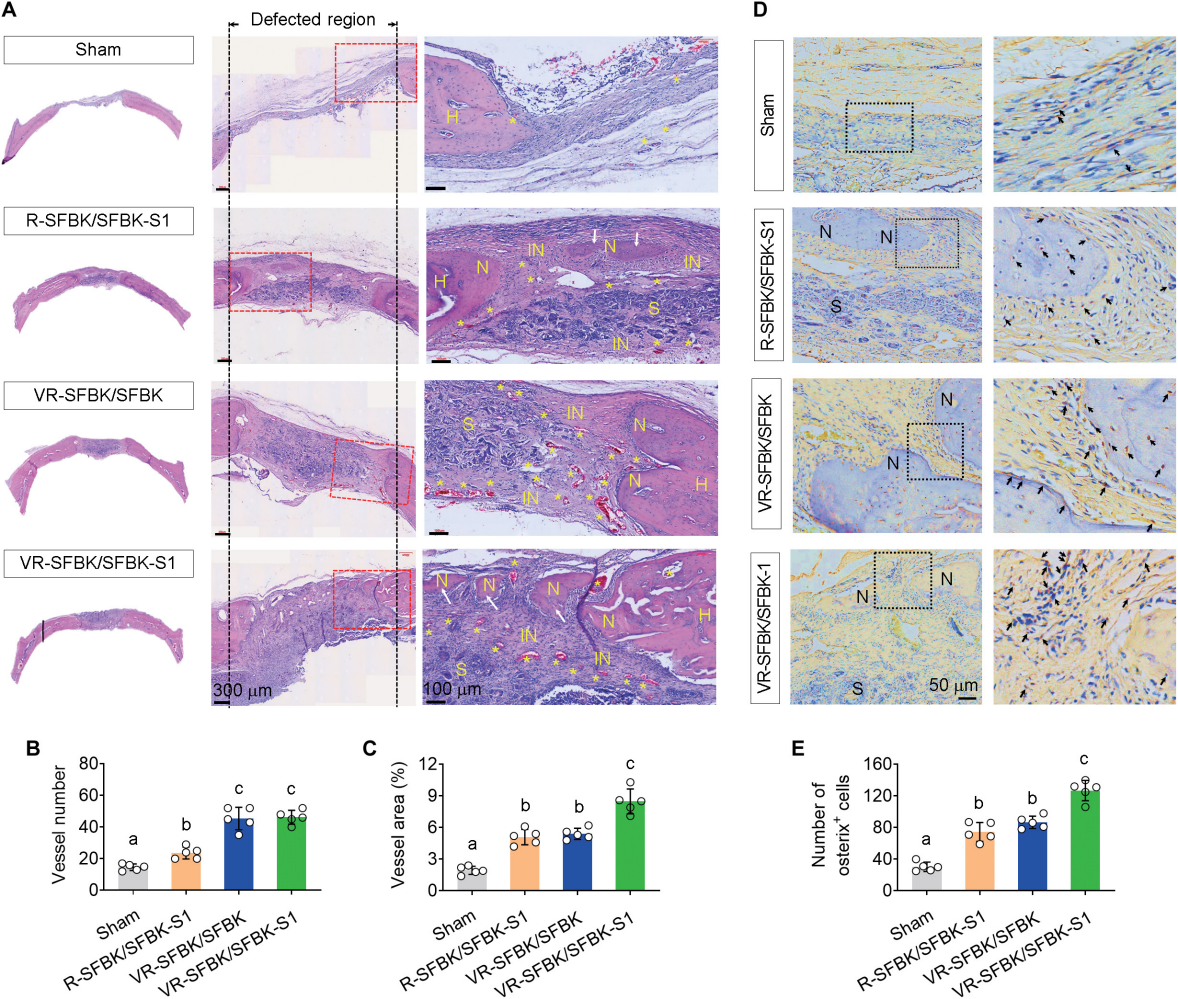
Involvement of new blood vessel formation and osteogenic factor expression in bone healing enhanced by puzzle fitting of scaffolds. (A) H&E-stained histological images of calvarial defects including defect regions at 12 weeks postimplantation. (B) The numbers and (C) area (%) of blood vessels formed in the defective regions. (D) Immunohistochemical (IHC) images showing the expression pattern of osterix in calvarial defects 12 weeks after implantation with the indicated scaffolds. (E) Quantitative comparison of osterix-positive cells among the groups. The letters a to c indicate significant differences (*P* < 0.05) among the groups from ANOVA followed by Tukey’s multiple-comparisons test (*n* = 5). H, host bone; N, new bone; IN, immature bone; S, scaffold.

As the μCT analysis indicated a crucial role of vasculogenic induction in SFBK scaffold-enhanced bone healing, we further evaluated the relationship between the formation of new bones and blood vessels at the defects by H&E staining. The sham group exhibited that a portion of the overlying tissue collapsed into the defect space, but all groups implanted with the puzzle-fitted scaffolds showed a well-maintained structure of scaffolds at defect regions (Fig. 7A). Compared with the sham group, all scaffold-implanted groups had greater new bone and blood vessel (indicated with yellow stars) formation. Specifically, the R-SFBK/SFBK-S1 group induced more new bone formation in the center of scaffolds (marked with white arrows) than the VR-SFBK/SFBK group, and induction was further apparent in the VR-SFBK/SFBK-1 group. When the number and area of newly formed blood vessels were evaluated, both the VR-SFBK/SFBK and VR-SFBK/SFBK-S1 groups showed markedly higher values than the R-SFBK/SFBK-S1 group (Fig. 7B and C). In addition, our IHC results supported the correlation of puzzle-fitted scaffold-stimulated healing of bone defects with the expression of osterix, the osteogenic transcription factor (black arrows) (Fig. 7D and E). These results suggest that cross-linking with SDF-1 or in vivo vascularization accelerates the efficacy of SFBK scaffolds on bone healing and that efficacy is substantially augmented by combining the processes via puzzle fitting. These results also postulate that puzzle fitting with an SFBK-S1 scaffold stimulates bone healing in the center of defects via SDF-1-mediated recruitment of multipotent stem cells.

### Biological properties of newly formed bones in relation to vascularization, SDF-1 cross-linking, or both

**Fig. 8.**
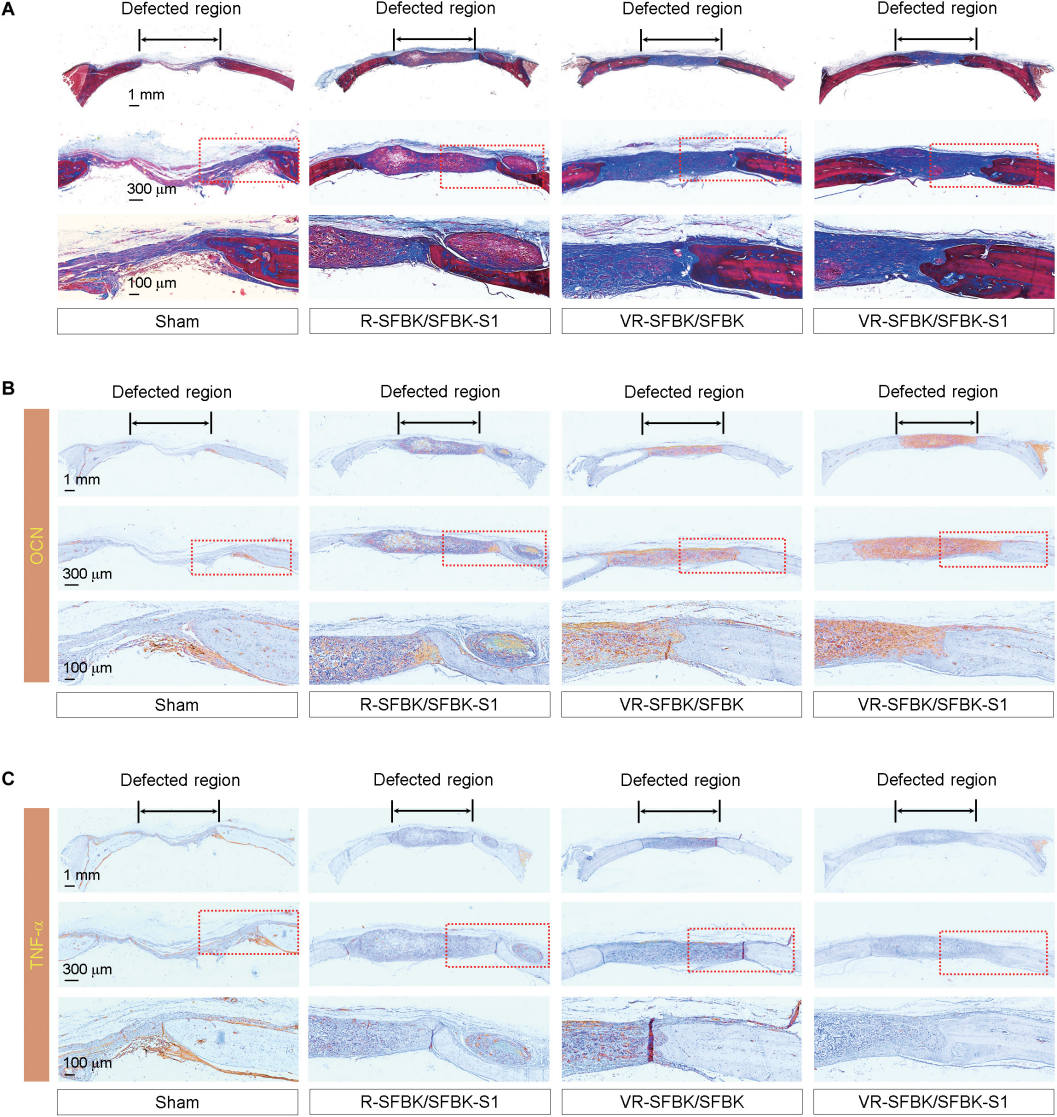
Biological properties of newly formed calvarial bones in relation to SDF-1 cross-linking, in vivo vascularization, or both. (A) Masson’s trichrome-stained histological images of calvarial defects including whole defect regions at 12 weeks postimplantation, in which collagen, bone, and nuclei are stained blue, red, and black to dark brown, respectively. IHC images showing the expression patterns of (B) osteocalcin (OCN) and (C) tumor necrosis factor-α (TNF-α) in calvarial defects 12 weeks after implantation with the indicated scaffolds. Representative results from 5 different samples are shown.

Collagen is a highly abundant protein in the extracellular matrix and is also a key component of bones. Controlled synthesis and the composition of collagens are very important in the formation, maturation, and strength of bones [36]. We performed Masson’s trichrome staining and evaluated the synthetic status of collagen and bone in the defective regions in relation to the SDF-1 cross-linking, vascularization, or both. Consistent with the results from H&E and μCT analyses, the quantity of newly formed bones was apparently higher in the groups implanted with the scaffolds compared with that in the sham group (Fig. 8A). Specifically, the composition of collagen in newly formed bones was greater in the VR-SFBK/SFBK and VR-SFBK/SFBK-S1 groups compared with that in the R-SFBK/SFBK-S1 and sham groups. These results suggest that in vivo vascularization of the scaffold rather than SDF-1 cross-linking is predominantly required for the synthesis of collagens, and this leads to the formation and maturation of new bones at defect regions. OCN is the most abundant noncollagenous and osteoblast-secreted protein in bone and participates in bone maturation and remodeling at the late stage of osteogenic differentiation [37]. The results from IHC staining supported a correlated expression of OCN with new bone formation at the defects, in which the VR-SFBK/SFBK-S1 group exhibited the greatest intensity specific to anti-OCN antibody among the experimental groups. Similar to the results shown in Fig. 7D, IHC staining of the sections including whole defective region also indicated greater expression of osterix at the defect regions implanted with VR-SFBK/SFBK or VR-SFBK/SFBK-S1 compared with that with R-SFBK/SFBK-S1 or with the sham group (Fig. S5). A surgical operation along with implantation with scaffolds can provoke inflammatory activation, and that activation disturbs the process required for bone healing and causes necrotic cell death in and around the defective sites. Among various inflammatory mediators, TNF-α is a well-known cytokine capable of affecting inflammatory response and the biocompatibility of scaffolds implanted for tissue regeneration. Regarding this, the strategy to inhibit the expression of TNF-α or TNF-α-mediated inflammatory activation can improve the biocompatibility and performance of scaffolds [38]. When the presence of TNF-α at the defect sites was evaluated by IHC staining, all of the scaffold-implanted groups revealed lower expression of the cytokine compared with the sham group (Fig. 8C). Importantly, the R-SFBK/SFBK-S1 and VR-SFBK/SFBK-S1 groups barely expressed TNF-α at the defects, whereas the VR-SFBK/SFBK group showed a slight expression of the cytokine in the implanted region. These results indicate that the implanted scaffolds did not cause inflammatory activation at defect regions but rather can suppress the expression of TNF-α in relation to cross-linking with SDF-1. It is important to note that SDF-1 is a chemokine that can be used in scaffolds to promote the healing of defect tissues, as well as to suppress inflammatory response during the healing process [39,40]. Therefore, we consider that the SFBK-based scaffolds did not cause cytotoxicity in in vitro and in vivo conditions, and SDF-1-enhanced bone healing is orchestrated by its potency to inhibit inflammation and to recruit multipotent stem cells.

The process of bone healing involves complicated biological events such as the migration and differentiation of cells, release of cytokines and growth factors, and the formation of new blood vessels, and that process is tightly affected by the mechanical and biochemical environments of scaffolds [41]. Our findings demonstrate that in vivo vascularization enhances bone healing by forming new blood vessels, and this process in combination with SDF-1 synergistically facilitates the healing of bone defects. Our results support that cross-linking with SDF-1 enhances new bone formation at the defects by stimulating the recruitment of multipotent stem cells toward the center of puzzle-fitted scaffolds, as well as by suppressing the inflammatory response at the defects. The current findings also suggest that the in vivo vasculogenic process efficiently induces the formation of new blood vessels and facilitates balanced bone formation and maturation at large bone defects [42]. In addition, our results indicate that BK-contained scaffolds contribute to desirable tissue engineering and hard-tissue regeneration via the sustained and controlled release of calcium ions [43]. Taken together, we postulate that in addition to the biocompatible microenvironment of SFBK scaffolds, BK-released calcium ions, SDF-1-mediated cell recruitment and anti-inflammation, and Ang1-stimulated angiogenesis and collagen synthesis might orchestrate the behaviors of cells retained and/or around the implanted scaffolds, eventually leading to synergistic enhancement of bone regeneration. However, it should be considered that our present study contains some limitations such as the complicated surgical procedures for in vivo vascularization and implantation and the relatively lower efficacy of the fabricated scaffolds in repairing critical-sized calvarial defects. Strategies to induce nonsurgical formation of blood vessels at defects, as well as to improve the performance of scaffolds on the healing of large bone defects, are required for further application of the biocompatible SFBK scaffolds. Regarding this, we suggest that a direct implantation with SFBK scaffolds conjugated with cartilage oligomeric matrix protein–Ang1 instead of native Ang1 together with bone morphogenetic protein 2 and SDF-1 may result in better and completed healing of large bone defects than do the vascularized and/or SDF-1-cross-linked SFBK scaffolds [19,30,33].

## Conclusion

We propose a novel modular assembly strategy that fits a round SFBK scaffold into the center of a VR-SFBK scaffold. In this study, we induced in vivo vascularization and stem cell recruitment using SFBK looping around blood vessels and the puzzle-fitting process. We investigated the efficacy on bone regeneration in relation to in vivo vascularization, SDF-1 cross-linking, or both. This study demonstrated that an SDF-1-cross-linked scaffold may recruit multipotent stem cells through newly formed blood vessels after puzzle fitting with a VR-SFBK scaffold and may induce anti-inflammation and balanced regeneration of new bones from the center to the edge of defects. The beneficial effects of the puzzle-fitting process on bone regeneration may involve the formation of microvascular networks through microsurgical anastomosis and immediate perfusion to increase stem cell mobilization. Overall, this study supports that in addition to in vivo neovascularization and stem cell recruitment, BK-released calcium ions are in part associated with bone regeneration at defects. However, further efficient strategies to avoid a complicated surgical operation and to improve the healing of large bone defects are required for the clinical application of SFBK-based scaffolds.

## Ethical Approval

This study used animals strictly following the recommendations in the Guide for the Animal Care and Use of Jeonbuk National University. All of the protocols applied in this study were approved by the University Committee on Ethics in the Care and Use of Laboratory Animals (JBNU 2021-0146).

## Data Availability

The data that support the findings of this study are available from the corresponding author upon reasonable request.
