# Supplementary material for: A Novel Strategy to Enhance the Bone Healing Efficacy of Composite Scaffolds via Induction of Cell Recruitment and Vascularization
Source: Biomater Res. 2025 Apr 10;29:0185. doi: 10.34133/bmr.0185 (PMC11982616; doi:10.34133/bmr.0185)
Supplement: Supplementary 1 — Figs. S1 to S5 Video S1 [file bmr.0185.f1.zip › Supplementary materials.docx]

**Supplementary materials**

**A Novel Strategy to Enhance the Bone Healing Efficacy of Composite Scaffolds via Induction of Cell Recruitment and Vascularization**

**Supplementary Figures and legends**


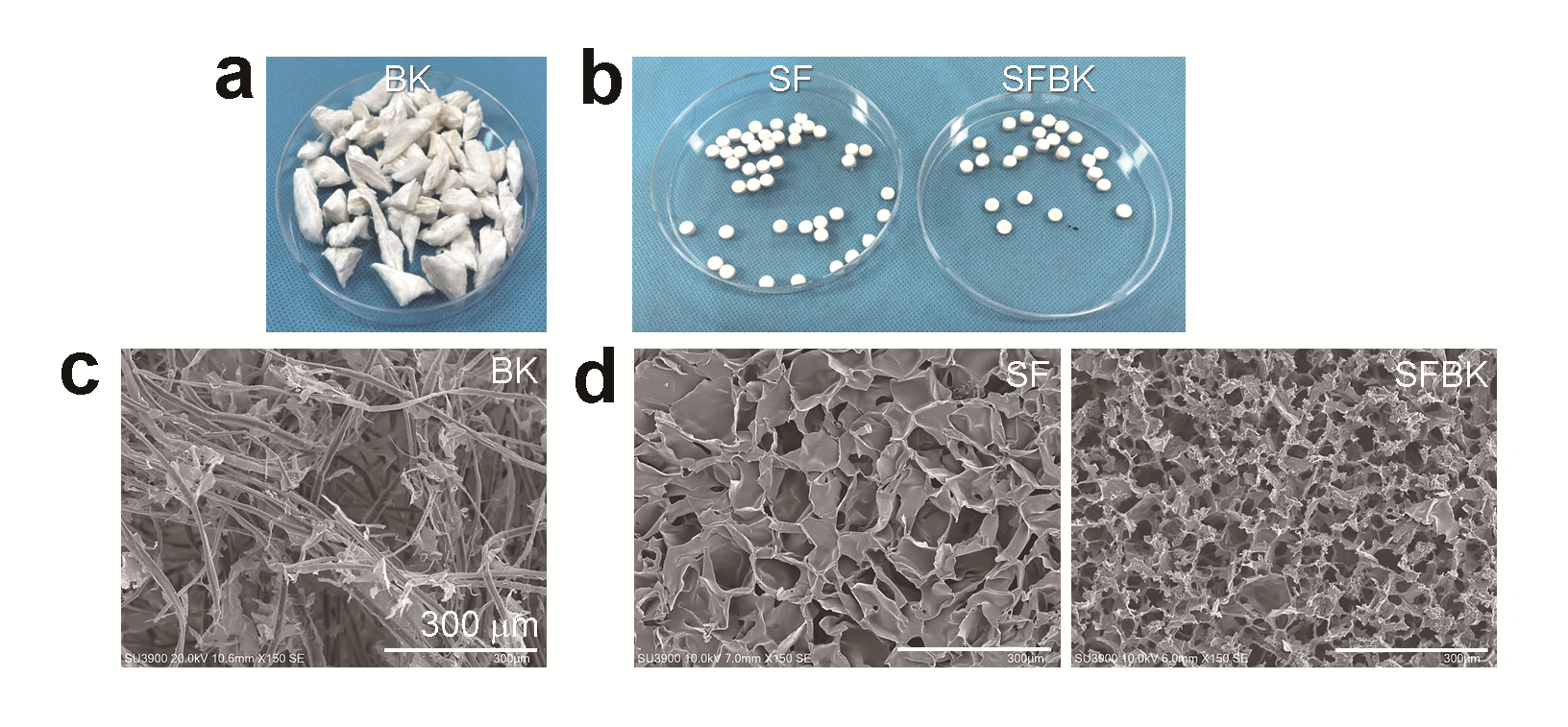


**Figure S1.** Digital photos of a) the lyophilized BK mass and b) the fabricated SF and SFBK scaffolds together with c and d) their SEM images.


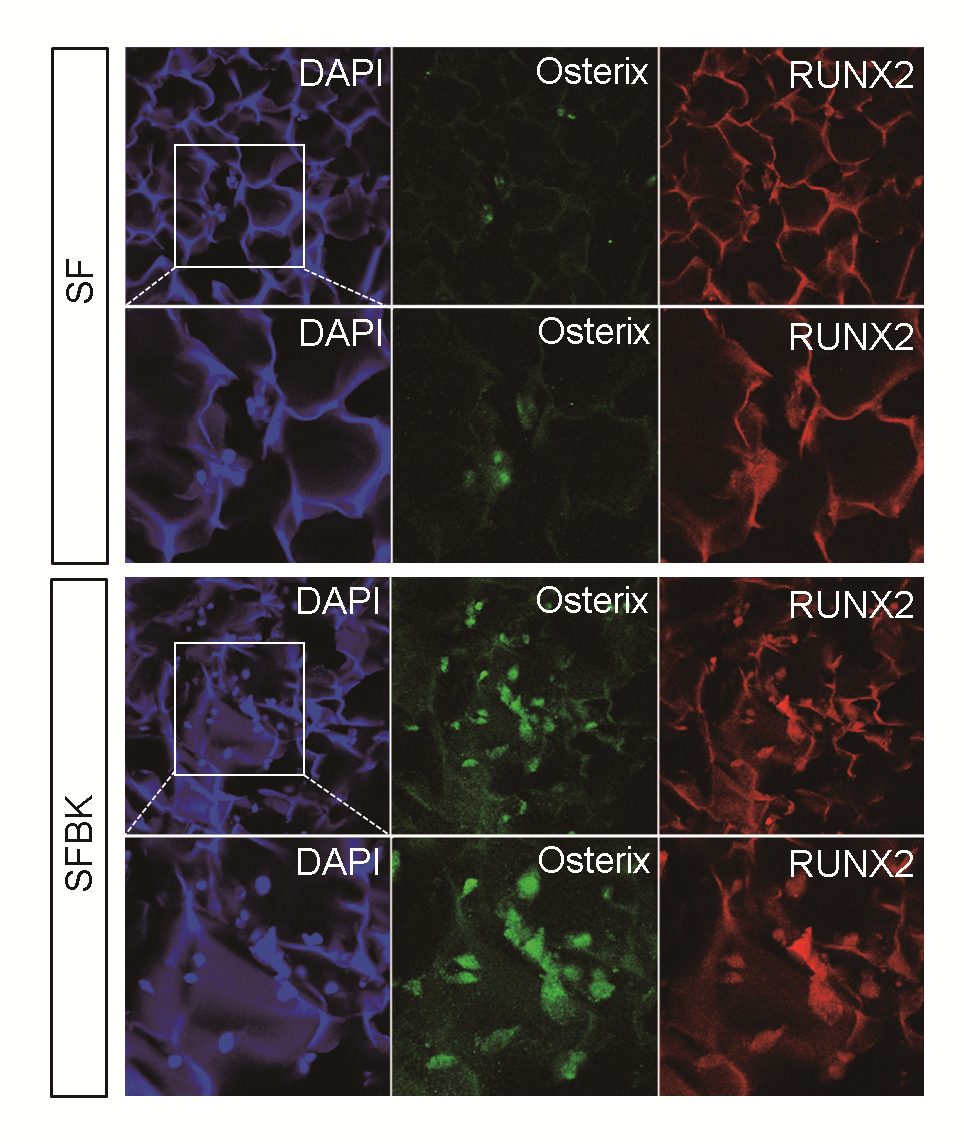


**Figure S2.** Osterix- and RUNX2-specific individual immunofluorescence images of hMSCs cultured for five days on SF or SFBK scaffold.


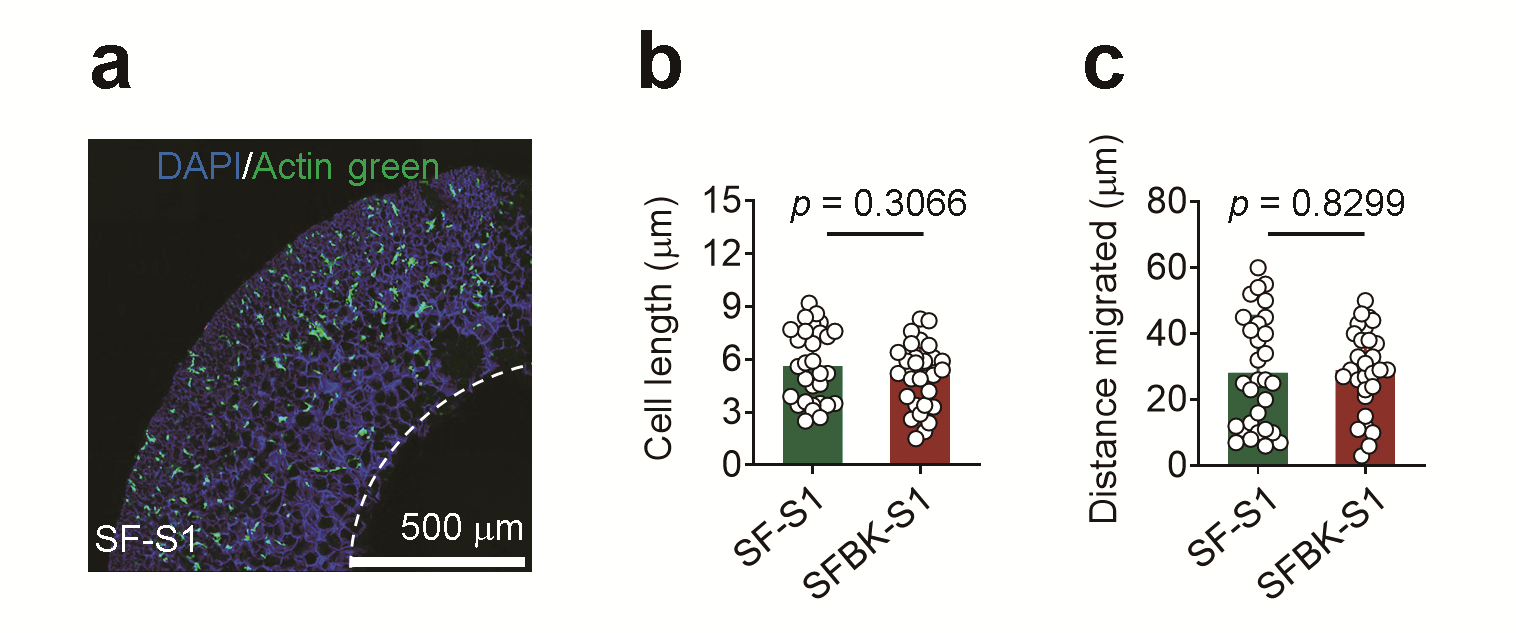


**Figure S3.** a) CLSM image showing the migration of hMSCs on SDF-1-linked SF scaffold (SF-S1) along with the quantitative comparison of b) cell length and c) migration distance of the cells with them cultured on SFBK-S1 scaffold. The *p*-value was determined by unpaired Student’s *t*-test with parametric and Welch’s correction.


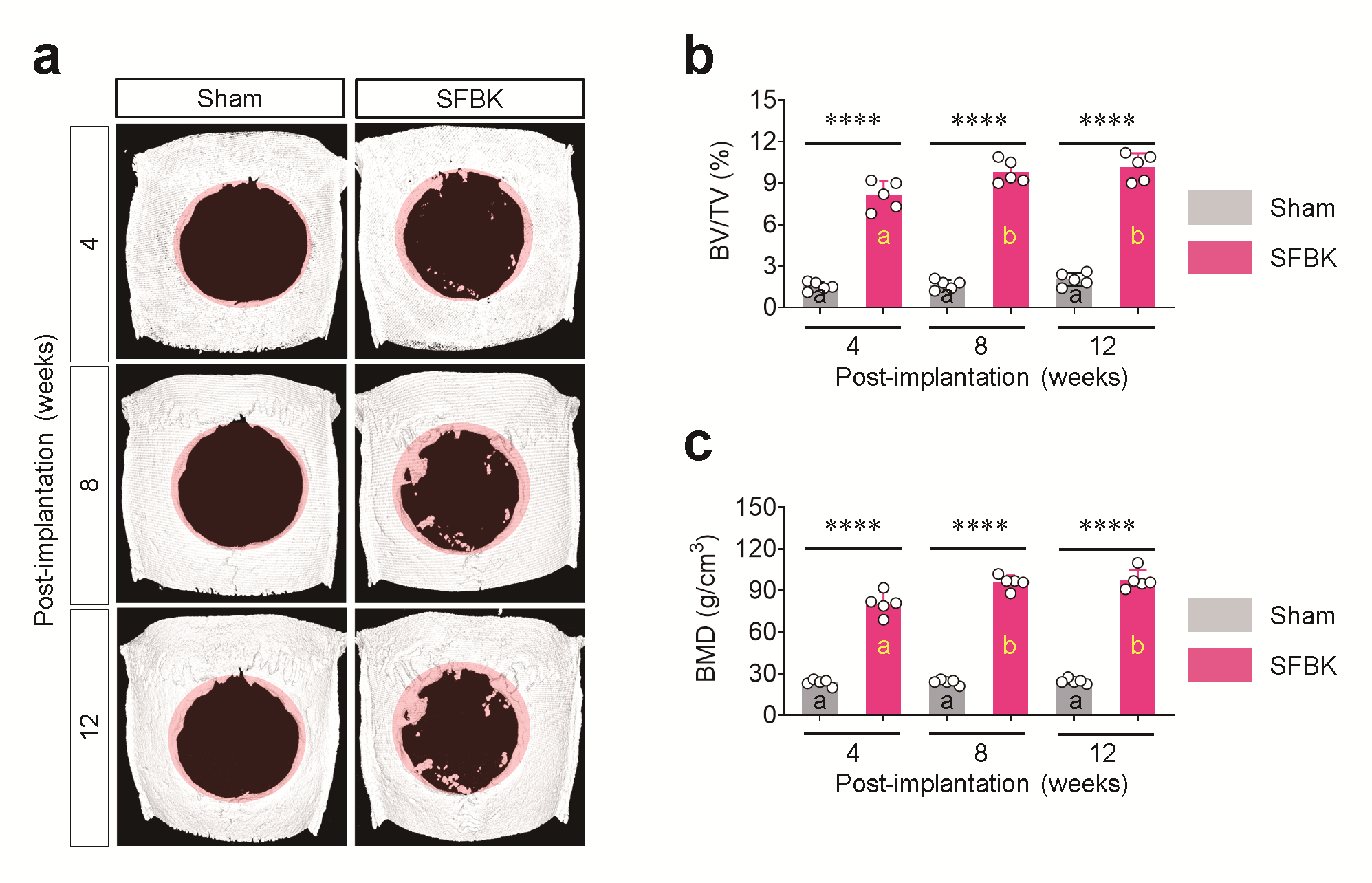


Figure S4. Comparison of bone healing efficacies in the rats implanted without (sham) and with SFBK alone. a) The μCT images showing calvarial bones including defect regions of the sham and SFBK groups. The values of b) BV/TV (%) and c) BMD (mg/cm^3^) were determined at four, eight, and 12 weeks post–implantation. The superscripts^(a and b)^ indicate significant differences (*p* < 0.05) from ANOVA followed by Tukey’s multiple comparisons test in relation to the weeks after implanation within the same group (*n* = 5). *****p* < 0.0001 by unpaired Student’s *t*-test with parametric and Welch’s correction.


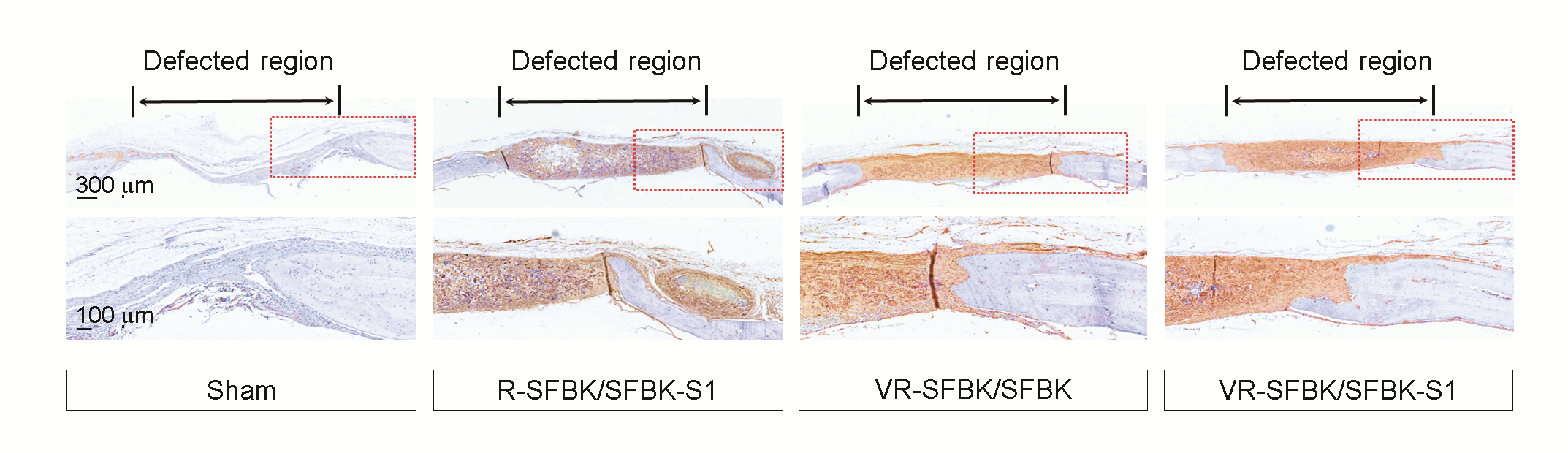


**Figure S5.** IHC images showing the expression pattern of osterix in the sections including whole defect region 12 weeks after implantation with the indicated scaffolds. Representative results from five different samples are shown.
