# Supplementary figures and images for: A Novel Strategy to Enhance the Bone Healing Efficacy of Composite Scaffolds via Induction of Cell Recruitment and Vascularization
Source: Biomater Res. 2025 Apr 10;29:0185. doi: 10.34133/bmr.0185 (PMC11982616; doi:10.34133/bmr.0185)

## Slide 1
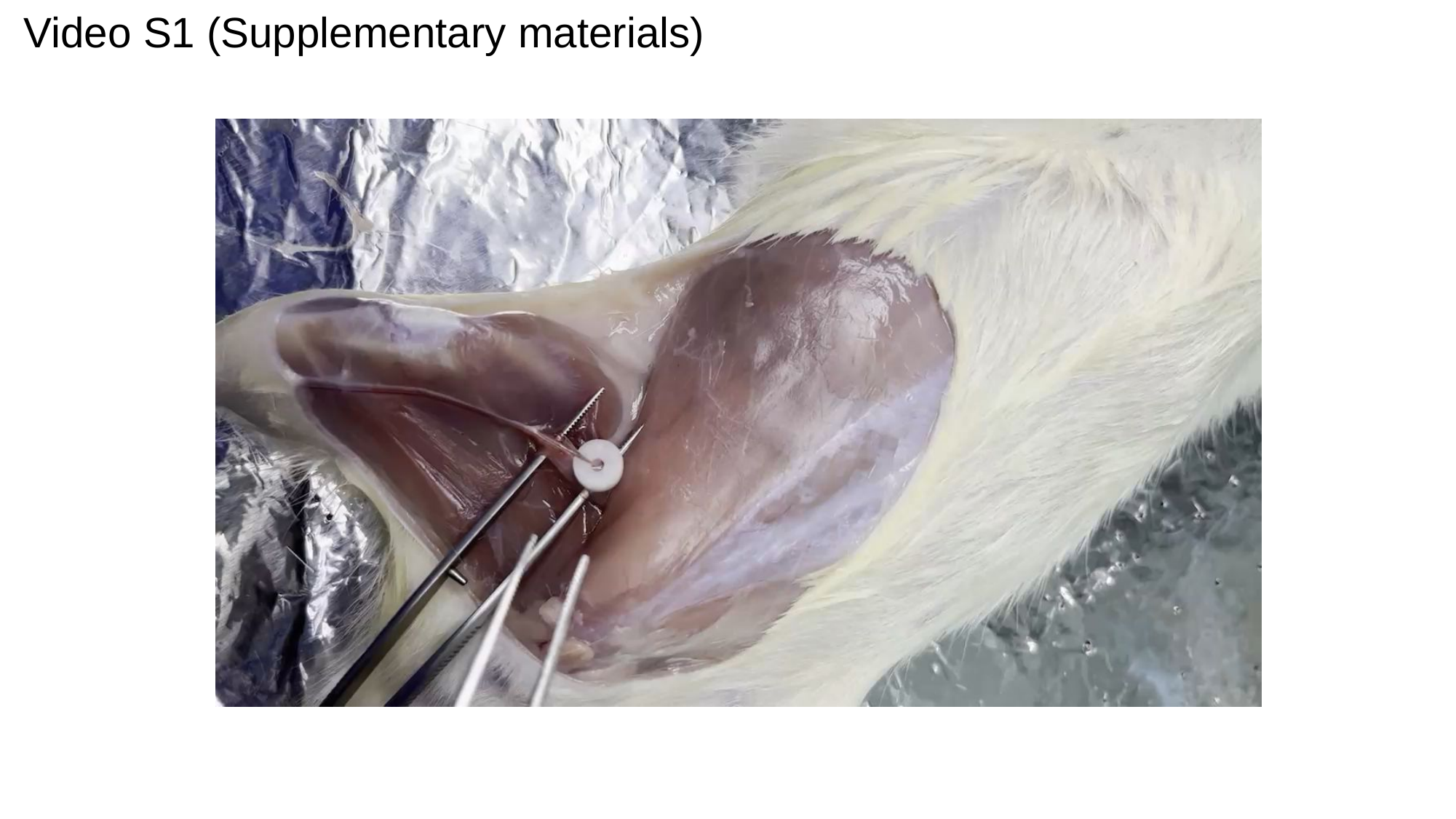

Video S1 (Supplementary materials)

Supplement: Supplementary 1 — Figs. S1 to S5 Video S1 [file bmr.0185.f1.zip › Supplementary materials (video 1).pptx]
